# HMGB1, a pathogenic molecule that induces neurite degeneration via TLR4-MARCKS, is a potential therapeutic target for Alzheimer’s disease

**DOI:** 10.1038/srep31895

**Published:** 2016-08-25

**Authors:** Kyota Fujita, Kazumi Motoki, Kazuhiko Tagawa, Xigui Chen, Hiroshi Hama, Kazuyuki Nakajima, Hidenori Homma, Takuya Tamura, Hirohisa Watanabe, Masahisa Katsuno, Chiemi Matsumi, Masunori Kajikawa, Takashi Saito, Takaomi Saido, Gen Sobue, Atsushi Miyawaki, Hitoshi Okazawa

**Affiliations:** 1 Department of Neuropathology, Medical Research Institute, Tokyo Medical and Dental University, 1-5-45 Yushima, Bunkyo-ku, Tokyo 113-8510, Japan; 2 Laboratory for Cell Function Dynamics, Brain Science Institute, RIKEN, 2-1 Hirosawa, Wako, Saitama 351-0198, Japan; 3 Department of Bioinformatics, Institute of Bioinformatics, Soka university, 1-236 Tangi-machi, Hachioji, Tokyo 192-8577, Japan; 4 Department of Neurology, Nagoya University Graduate School of Medicine, 65 Tsurumai-cho, Showa-ku, Nagoya 466-8550, Japan; 5 Medical and Biological Laboratories Co., LTD. 4-5-3 Sakae, Naka-ku, Nagoya 460-0008, Japan; 6 Laboratory for Proteolytic Neuroscience, Brain Science Institute, RIKEN, 2-1 Hirosawa, Wako, Saitama 351-0198, Japan; 7 Center for Brain Integration Research, Tokyo Medical and Dental University, 1-5-45 Yushima, Bunkyo-ku, Tokyo 113-8510, Japan

## Abstract

Alzheimer’s disease (AD) is the most common neurodegenerative disease, but it remains an intractable condition. Its pathogenesis is predominantly attributed to the aggregation and transmission of two molecules, Aβ and tau; however, other pathological mechanisms are possible. Here, we reveal that phosphorylation of MARCKS, a submembrane protein that regulates the stability of the actin network, occurs at Ser46 prior to aggregation of Aβ and is sustained throughout the course of AD in human and mouse brains. Furthermore, HMGB1 released from necrotic or hyperexcitatory neurons binds to TLR4, triggers the specific phosphorylation of MARCKS via MAP kinases, and induces neurite degeneration, the classical hallmark of AD pathology. Subcutaneous injection of a newly developed monoclonal antibody against HMGB1 strongly inhibits neurite degeneration even in the presence of Aβ plaques and completely recovers cognitive impairment in a mouse model. HMGB1 and Aβ mutually affect polymerization of the other molecule, and the therapeutic effects of the anti-HMGB1 monoclonal antibody are mediated by Aβ-dependent and Aβ-independent mechanisms. We propose that HMGB1 is a critical pathogenic molecule promoting AD pathology in parallel with Aβ and tau and a new key molecular target of preclinical antibody therapy to delay the onset of AD.

Therapeutic strategies to treat Alzheimer’s disease (AD)^1^ have been tested in clinical trials and have shown insufficient results. Treating patients with anti-Aβ antibodies and γ-secretase inhibitors after the onset of dementia was ineffective^2^,^3^. The results have shifted research interests to the earliest molecular events in the AD brain^4^,^5^, while the evaluation and discussion of clinical trials remain ongoing^6^. Treating pre-clinical AD patients with the same anti-Aβ antibodies and γ-secretase inhibitors may overcome the failure of previous clinical trials^7^. At the same time, focusing on the earliest pathology of AD may identify other pathological mechanisms that have not received sufficient attention in previous studies due to the focus on aggregation of Aβ and tau^8^ and that might be more effective as the targets of therapeutics. These mechanisms could include various molecular events at the stage when Aβ concentration increases and early Aβ oligomerization occurs but before Aβ fibrils aggregate in the brains of AD patients^4^.

In accordance with this idea, we carried out a comprehensive phosphoproteome analysis of brain tissue samples from mouse AD models and human AD patients^9^. Selecting molecules whose abnormal phosphorylation was shared by multiple AD models, we identified 17 proteins that may play critical roles in the early stage of AD pathology. Interestingly, the phosphorylation state of most of the proteins in the cerebral tissues of human AD patients was changed. Notably, the phosphorylation of MARCKS (Myristoylated alanine-rich C-kinase substrate) was initiated at the earliest time point (1 month of age) in the mouse model prior to Aβ aggregation as determined by immunohistochemistry and before the onset of cognitive impairment^9^. MARCKS is a submembrane protein anchoring actin cytoskeleton network and a representative substrate of protein kinase C (PKC)^10^. However, the phosphoproteome analysis was based on integrating the values of all of a protein’s phosphorylation sites. Thus, further investigation was necessary to clarify the details of pathological cell signalling mediated by each phosphorylation site in the early stages of AD.

In this study, we focus on MARCKS and dissect biological significance of phosphorylation at Ser46 that is shared between mouse AD models and human AD patients. We reveal that the phosphorylation at Ser46 decreases the affinity between MARCKS and actin, destabilizes dendritic spines, and degenerates neurites. We also reveal that HMGB1, which is well known as a critical intracellular molecule regulating DNA architecture^11^,^12^, DNA damage repair^13^, transcription and autophagy^12^,^14^, as well as an important extracellular DAMP (damage associated molecular pattern) molecule^15^, is released from hyper-excitatory neurons, binds to a DAMP receptor TLR4 (Toll-like receptor 4)^16^, and triggers MARCKS phosphorylation at Ser46 in the downstream of the signal pathway. Subcutaneous injection of anti-HMGB1 monoclonal antibody inhibits neurite degeneration, stabilizes spines, and improves cognitive impairment in AD model mice. The phenotype improvements occur without affecting Aβ aggregation *in vivo,* since HMGB1 basically suppresses Aβ aggregation *in vitro*. These findings in this study indicate the significance of HMGB1 in AD as a pathogenic mediator and as a critical therapeutic target.

## Results

### MARCKS phosphorylation at Ser46 is a hallmark of neurite degeneration

**Figure 1:**
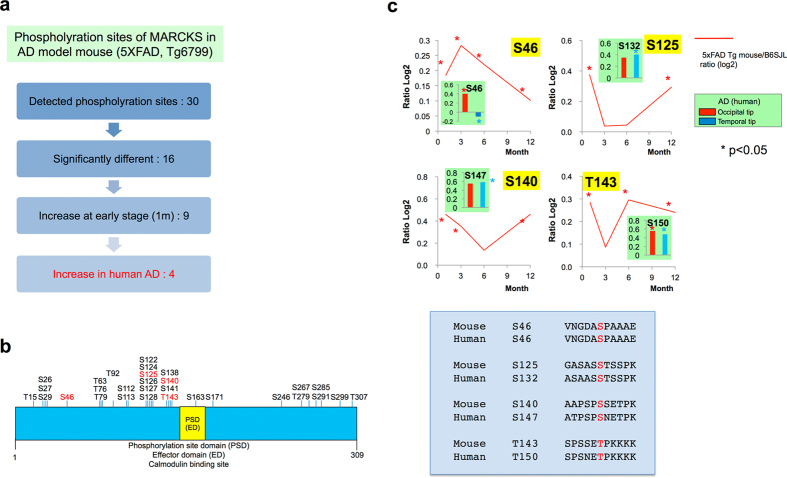
Ser46 phosphorylation of MARCKS is altered in a mouse model and in human postmortem brains. **(a)** Strategy to select critical phosphorylation sites that are hyperphosphorylated at 1 month of age prior to amyloid aggregation in the cortex of 5xFAD mice (line: Tg6799); similar changes were also observed in postmortem human brain tissues (temporal or occipital lobe samples). The selected phosphorylation sites are candidates that are altered at the earliest stage and continue until the end stage of AD pathology. **(b)** Schematic presentation of mass spectrometry-detected (black and red letters) phosphorylation sites and mouse and human (red letters) phosphorylation sites of MARCKS. **(c)** Chronological changes in the phosphorylation ratio (log_2_) at selected sites by mass spectrometric analysis and their changes in postmortem human AD cortex samples (temporal and occipital samples) at the corresponding phosphorylation sites. The lower panel shows the comparison of amino acid sequences near the corresponding phosphorylation sites between mice and humans.

In the phosphoproteome analysis^9^, we identified 30 phosphorylation sites of MARCKS in the 5xFAD mouse AD model (line Tg6799)^17^ (Fig. 1). In this study, we further investigated the phosphoproteome data. First, we selected candidates with critical phosphorylation sites using a flow chart (Fig. 1a). Among the 30 phosphorylation sites of MARCKS, 16 sites were significantly altered compared to proteins from wild type mice with the same genetic background (B6/SJL); 9 of the 16 sites were hyperphosphorylated at 1 month of age before Aβ aggregation, and 4 of the 9 sites were also hyperphosphorylated in postmortem human brains of AD patients compared to non-neurological disease patients (Fig. 1a).

The four phosphorylation sites (Ser46, Ser125, Ser140, Thr143) were different from the well-characterized sites near the PSD/ED domain (Ser159, Ser163, Ser170) that are phosphorylated by PKC to regulate the anchoring of MARCKS to the cell membrane^18^,^19^,^20^ or to the myristoylated N-terminus^21^ (Fig. 1b). Additionally, our phosphoproteome data did not indicate any changes in Ser163 in the AD mouse model, and Ser159 and Ser170 were not identified (Fig. 1b). Therefore, we focused on the four sites (Ser46, Ser125, Ser140, Thr143) that were hyperphosphorylated prior to Aβ aggregation in the mouse models and whose corresponding sites in human AD patients (Ser46, Ser132, Ser147, Thr150, respectively) were phosphorylated in the final stages of disease (Fig. 1c).

**Figure 2:**
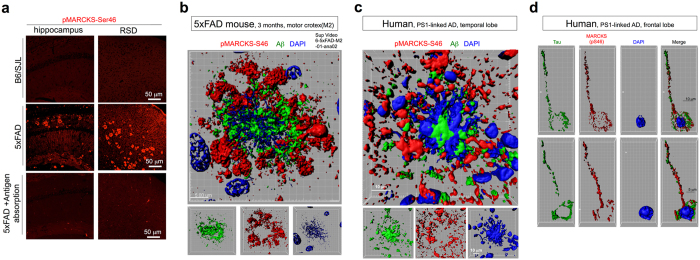
Ser46 phosphorylation of MARCKS is a marker of degenerative neurites. **(a)** Anti-pMARCKS (Ser46)-stained amyloid plaque-like structures in the cerebrum of 5xFAD mice but not in wild type B6/SJL mice. The synthetic peptide ENGHVKVNGDA(pS)PA was used for antigen absorption. **(b)** Three-dimensional reconstruction of Aβ (green), Ser46-pMARCKS (red), and DAPI (blue) co-staining revealed that Ser46-phosphorylated MARCKS surrounds amyloid aggregates after cell death. The corresponding movie is attached as Supplementary Information. **(c)** Three-dimensional reconstruction of Aβ (green), Ser46-pMARCKS (red), and DAPI (blue) revealed similar patterns in human AD brains. **(d)** Three-dimensional reconstruction of tau (green), Ser46-pMARCKS (red), and DAPI (blue) revealed that Ser46-phosphorylation of MARCKS occurs in the axons of human AD brains.

First, we synthesized phospho-peptides corresponding to the four sites and generated polyclonal antibodies. We next performed immunohistochemistry using 5xFAD mice (line Tg6799). Surprisingly, the anti-phospho-Ser46-MARCKS antibody raised against the synthetic peptide ENGHVKVNGDA(pS)PA, but not the other antibodies, detected amyloid plaque-like patterns at a low magnification (Fig. 2a, Supplementary Figure 1). Characterization of this antibody by Western blot revealed specific bands of MARCKS-EGFP or EGFP-MARCKS expressed in HeLa cells (Supplementary Figure 2, coloured arrows) and three non-specific bands (Supplementary Figure 2, black arrows). The specific bands, but not the non-specific bands, were absorbed with the antigen peptide (Supplementary Figure 2). Given that the amyloid plaque-like pattern in immunohistochemistry was absorbed by the synthetic peptide (Fig. 1a), these patterns suggested that Ser46 phosphorylation of MARCKS occurs within or around amyloid plaques. Notably, several non-specific bands were also observed with a commercial antibody against MARCKS (Supplementary Figure 2).

To exclude the possibility of a cross-reaction between the anti-phospho-Ser46-MARCKS antibody and Aβ, we used cerebral tissues of 5xFAD mice and examined the relationship between Aβ and pSer46-MARCKS staining at a higher magnification with 3D images (Fig. 2b, Supplementary Figure 3, Supplementary Video 1). The results were very clear and showed that the pSer46-MARCKS stains were distinct from Aβ stains. Instead, we frequently observed that the pSer46-MARCKS staining surrounded Aβ aggregation (Fig. 2b, Supplementary Figure 3, Supplementary Video 1). Similar patterns were also observed in human familial AD brains carrying a PS1 gene mutation (M146L) (Fig. 2c, Supplementary Figure 4, Supplementary Video 2). 3D images of transparent brain tissues of human mutant APP knock-in mice^22^ using the Sca*l*eS method^23^ also confirmed the close relationship between Aβ and pSer46-MARCKS from 8 to 20 months of age (Supplementary Figure 5a, Supplementary Video 3a–c).

Although Aβ has been reported to promote MARCKS phosphorylation in microglia^24^,^25^, co-staining with a microglial marker (MRF1) distinguished pSer46-MARCKS from microglia (Supplementary Figure 6a). Instead, we confirmed that neurons were located at the centre of pSer46-MARCKS-positive neurites (Supplementary Figure 6b).

We next examined brain regions without extracellular Aβ aggregation in the cerebral cortex of human PS1-linked AD patients and found that tau and pSer46-MARCKS were co-localized in axon fibres (Fig. 2d); 3D images further supported this finding (Supplementary Video 4). We also found that pSer46-MARCKS localized to the dendrites of neurons (Supplementary Figure 3). These unique relationships among pSer46-MARCKS, Aβ aggregation, and axon/dendrite markers suggest that pSer46-MARCKS phosphorylation may occur in degenerative neurites/regions of neuritic dystrophy surrounding the Aβ plaques, which are classical hallmarks of AD both in human and mouse model pathologies^26^,^27^ and in impaired dendrites^28^.

Another notable observation was a collapsing nucleus faintly stained with DAPI that was frequently located at the centre of extracellular Aβ aggregation both in the AD mouse model and human AD patients (Fig. 2, Supplementary Figures 3 and 4). Given the hypothesis that neurons with intracellular Aβ accumulation die, leaving Aβ aggregation^29^,^30^,^31^, this finding further suggests that the pSer46-MARCKS antibody stained degenerating neurites that were previously connected to dying neurons.

### MARCKS phosphorylation by ERK/JNK leads to neurite degeneration

Next, we determined the functional significance of MARCKS phosphorylation at Ser46 in neurons. A previous study suggested that MARCKS is a sub-membrane protein that anchors the actin network to PIP_2_^10^, and its phosphorylation regulates dendritic spine morphology^21^, likely by altering the connection of the actin cytoskeleton network to PIP_2_ in the plasma membrane^18^,^19^,^20^. Our previous study showed that inhibitors of PKC, a MARCKS kinase, blocked the decrease in dendritic spines of neurons in 5xFAD mice at 3 months of age when extracellular Aβ aggregation begins^9^. These results prompted us to examine the role of MARCKS phosphorylation at Ser46 in dendritic spine dynamics.

**Figure 3:**
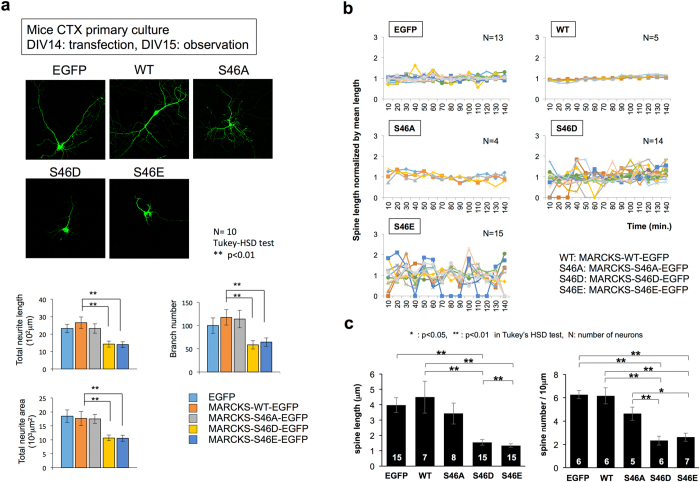
Ser46 phosphorylation of MARCKS impairs neurites and dendritic spines. **(a)** Expression of MARCKS mutants mimicking phosphorylated forms (S46E, S46D) decreased the total length, branching number, and total area of neurites of primary mouse cortical neurons, whereas expression of a MARCKS non-phosphorylated mutant (S46A) and the wild type did not affect these parameters. The lower graphs show the quantitative analyses. **(b)** Expression of MARCKS mutants mimicking phosphorylated forms (S46E, S46D) increased the instability of dendritic spines of primary mouse cortical neurons, whereas expression of a MARCKS non-phosphorylated mutant (S46A) did not. **(c)** Quantitative analyses of the length and density of the dendritic spines.

For this purpose, we constructed mutants of full-length MARCKS fused to EGFP: the phosphorylated mimics S46D and S46E and the non-phosphorylated mutant S46A. The constructs were transfected into mouse primary cortical neurons to observe their effects on the interaction between MARCKS and actin (Supplementary Figure 7). Co-precipitation assays revealed a weak interaction between actin and the S46D and S46E mutants (Supplementary Figure 7). We also found that the expression of S46D and S46E, but not S46A, decreased the neurite length, branching number and neurite area in mouse primary cortical neurons (Fig. 3a); destabilized dendritic spines (Fig. 3b); and decreased the spine length and density (Fig. 3c), consistent with the reported observations of AD pathology^32^,^33^,^34^.

Next, we determined the upstream signal leading to MARCKS phosphorylation at Ser46. The popular algorithm NetworKIN 3.0 (URL: http://networkin.info) indicated that MAPK1/2 (ERK2/1) and PKD1 (PKC mu) were candidate kinases (Supplementary Figure 8a). To confirm these predictions, we performed an *in vitro* phosphorylation reaction of GST-MARCKS with candidate MAPKs, including JNK, which has been implicated in Alzheimer’s disease, and performed mass spectrometry to examine whether these kinases could actually phosphorylate MARCKS at Ser46 (Supplementary Figure 8b). The results of the *in vitro* phosphorylation experiment and subsequent mass spectrometric analysis revealed that MAPK1/2 and JNKs could phosphorylate MARCKS at Ser46 (Supplementary Figure 8b). MAPK1/2 and JNKs are downstream of Toll-like receptor (TLR) signalling^16^,^35^,^36^,^37^. DAMPs/PAMPs (damage-associated molecular patterns), such as Aβ and HMGB1, are released from damaged cells^38^,^39^,^40^ and are representative ligands of TLR. Taken together, these results suggest that Aβ and/or HMGB1 activates the signalling pathway, leading to MAPK1/2 and JNK through interactions with TLR or other Aβ receptors, such as NMDA receptors^41^,^42^, EphB2^43^, PirB^44^, PrP^c^^45^, and others.

### HMGB1 induces MARCKS phosphorylation via TLR4

From our morphological observation of 5xFAD mice, HMGB1 was localized to the cytoplasm in abnormal neurons with intracellular Aβ (Supplementary Figure 9a). Aβ remained aggregated at the core of the dying cells after neuronal necrosis (Fig. 2b,c, Supplementary Videos 1 and 2), whereas HMGB1 dispersed and did not co-aggregate with Aβ in the ghost neurons (Supplementary Figure 9a–c), suggesting that HMGB1 was released after the rupture of the neurons. Co-staining of Aβ and pSer46-MARCKS showed that NeuN-positive neurons had been in the centre of the plaques surrounded by degenerative neurites (Supplementary Figure 6b).

In addition, immunohistochemistry of pSer46-MARCKS along with Aβ staining at multiple ages of 5xFAD mice revealed that MARCKS phosphorylation occurred at 1 month of age (Supplementary Figure 10a,b), which was consistent with our previous observations from the phosphoproteome analysis^9^. At this age, intracellular Aβ accumulation, but not extracellular Aβ aggregation, had occurred. Intriguingly, cell bodies of the neurons and the neurite fibres were stained with the anti-Ser46-pMARCKS antibody, but they did not co-localize with Aβ (Supplementary Figure 10a,b). At 3 or 6 months of age, the pSer46-MARCKS signals were observed to surround the ghost cells of the intracellular Aβ aggregations or the relevant extracellular Aβ aggregations, respectively (Supplementary Figure 10b). Chronological observation of human mutant APP knock-in mice^22^ using the ScaleS method^23^ also revealed that MARCKS phosphorylation occurred locally adjacent to the Aβ plaques from 8 to 17 months of age while it dispersed in the cortex or other brain regions irrespective of Aβ plaques at 20 months (Supplementary Figure 5b).

Therefore, we first examined whether extracellular HMGB1 induced phosphorylation of MARCKS at Ser46 by adding purified HMGB1 to a primary culture of mouse cortical neurons (Supplementary Figure 11a). Western blot analysis revealed that MARCKS phosphorylation at Ser46 was induced 60 minutes after the addition of HMGB1 (Supplementary Figure 11a). The response occurred in a dose-dependent manner at 5 nM or higher concentrations (Supplementary Figure 11b), whereas no response was triggered by the negative control, BDNF (Supplementary Figure 11c). Next, we tested the effect of various Aβ species on the phosphorylation of MARCKS at Ser46 (Supplementary Figure 11d). Aβ oligomers weakly triggered phosphorylation of MARCKS at Ser46 in mouse cortical primary neurons, whereas monomers and fibrils/aggregates had no effect (Supplementary Figure 11d). Incubation of HMGB1 for 48 hours at 37 °C further enhanced the HMGB1-mediated phosphorylation of MARCKS at Ser46 (Supplementary Figure 11e). The addition of Aβ to the HMGB1 incubation (incubation of the mixture for 48 hours at 37 °C) weakened the activity, although it still triggered MARCKS phosphorylation at Ser46 (Supplementary Figure 11e).

Finally, we found that a TLR ligand (LPS-EB) induced similar phosphorylation of MARCKS at Ser46 (Supplementary Figure 11f) and that a TLR antagonist (LPS-RS) blocked the HMGB1-induced phosphorylation of MARCKS at Ser46 (Supplementary Figure 11g). TLR4 knockdown by TLR4-shRNA suppressed the HMGB1-induced MARCKS phosphorylation at Ser46 (Supplementary Figure 11h). These results suggest that TLR4, one of the dominant TLRs in neurons^46^,^47^, mediates the signalling from HMGB1 to MARCKS phosphorylation at Ser46.

**Figure 4:**
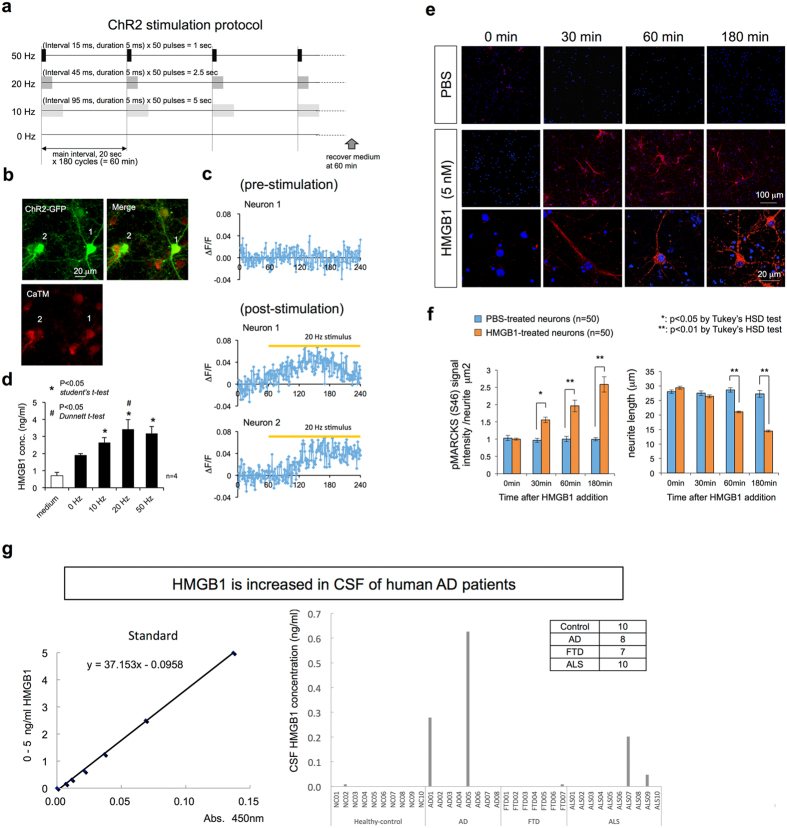
HMGB1 triggers Ser46 phosphorylation of MARCKS and is released from depolarized neurons. **(a)** The protocol to depolarize AAV-channelrhodopsin 2-GFP (ChR2)-infected mouse primary neurons. Wave length: 465-475 nm, light strength: 768–1056 lumens (=158730 lux in 9 cm × 7 cm area). **(b)** Images of neurons (9 × 10^4^ cells/well, 1.8 cm^2^/well, 2.4 ml of culture medium) that were expressing ChR2, in which the membrane depolarization-associated Ca^2+^ increase activated the CaTM-2-AM dye (Goryo Chemical, Inc.) in the cytoplasm. **(c)** Calculation of calcium signals from ChR2-expressing neurons. Pre- and post-stimulation conditions are shown with the duration of photo-stimulation. **(d)** Concentration of HMGB1 in the neurobasal medium (Thermo Fisher Scientific, Inc.). During the preparation of primary neurons, they were partially damaged as shown in (**a**), and HMGB1 was detected. The concentration was rapidly increased after photo-stimulation. **(e)** Addition of HMGB1 (5 nM in PBS) triggers Ser46-phosphorylation of MARCKS in the cytoplasm and shortening of the neurites in mouse primary cortical neurons, whereas PBS alone did not change the signal of Ser46-phosphorylated MARCKS or the neurite length of neurons. As damaged neurons are present in normal primary cultures, dot-like stains (not skein- or line-like stains) of Ser46-phosphorylated MARCKS were also found in nearly 10% of the neurons. **(f)** Quantitative analyses of Ser46-phosphorylated MARCKS signals and neurite lengths 180 minutes after the addition of HMGB1. **(g)** HMGB1 was increased in the cerebrospinal fluid (CSF) in a portion of the human AD patients. In addition, an increase was detected in ALS patients.

The reciprocal relationship of pSer46-MARCKS and intracellular Aβ by immunohistochemistry (Supplementary Figure 10b) suggested that intracellular Aβ accumulation does not directly trigger phosphorylation of MARCKS at Ser46. Instead, stimulation of primary cortical neurons with HMGB1 and/or Aβ (Supplementary Figure 11) indicated that extracellular HMGB1 and/or Aβ oligomers released from cells triggered phosphorylation of MARCKS at Ser46. HMGB1 is known to be released from necrotic cells^48^. Thus, neuronal necrosis, such as necrosis following intracellular Aβ accumulation^17^,^31^, is the first candidate mechanism to release HMGB1. As a second possible mechanism of extracellular release of HMGB1, we showed that hyperactivity of neurons could be a cause even before cell death (Fig. 4a–d). Photo-stimulation of mouse primary cortical neurons expressing channelrhodopsin 2 (ChR2) at a frequency of more than 10 Hz (Fig. 4a–c) increased the concentration of HMGB1 in the culture medium (Fig. 4d), suggesting an activity-dependent release of HMGB1 from neurons. This finding may be of interest considering that the default mode network, which has higher neuronal activity than the other brain regions, corresponds to the brain regions affected by Alzheimer’s disease in the early stages, as previously reported^49^.

When we added HMGB1 to primary cultures at a concentration of 5 nM, retraction of the pSer46-MARCKS-positive neurites occurred over a short time period (3 hours) (Fig. 4e,f). However, we could not detect obvious shrinkage of neurites by the addition of HMGB1 at concentrations lower than 1 nM (data not shown), which was consistent with the concentration needed for HMGB1 to induce MARCKS phosphorylation at Ser46 (Supplementary Figure 11b). The concentration needed for Aβ to induce MARCKS phosphorylation at Ser46 was much higher. At 25 nM, only the Aβ oligomer, but not the monomer or aggregate of Aβ, induced MARCKS phosphorylation at Ser46 (Supplementary Figure 11d). Considering the tendency of Aβ to remain aggregated and the rapid dispersion of HMGB1 from dying cells (Supplementary Figure 9a,b), we hypothesized that HMGB1 is the major factor inducing MARCKS phosphorylation and neurite degeneration.

### HMGB1 is increased in the extracellular fluid of human AD brains

The concentration of HMGB1 needed to trigger phosphorylation of MARCKS in primary cortical neuron cultures was 5 nM (125 ng/ml, Supplementary Figure 11b). The firing of primary neurons in the culture (9 × 10^4^ cells/well, 1.8 cm^2^/well, 2.4 ml of culture medium) increased the concentration of HMGB1 up to 1–2 ng/ml (Fig. 4d). Considering the neuronal concentration in the human brain (1.4 × 10^5^ cells/ml), our results predicted that such a hyperactive state would increase the concentration of HMGB1 to 4–8 ng/ml in the extracellular space of the brain, assuming that HMGB1 is diluted homogenously in the brain. Although this concentration is insufficient to trigger MARCKS phosphorylation, the local concentration at the synapse would be much higher and therefore may be sufficient. Additional factors, such as inflammation due to AD pathology, might increase the background concentration of HMGB1 in the extracellular space. Therefore, hyperactivity of neurons could be a risk factor for MARCKS phosphorylation in the AD brain. However, the discrepancy between the two concentrations (125 ng/ml vs. 8 ng/ml) could be considered a safeguard to prevent activity-dependent neurodegeneration in the physiological brain.

To test whether HMGB1 is increased in human AD tissues, we evaluated HMGB1 concentrations in the cerebrospinal fluid (CSF) of human AD patients by ELISA (Fig. 4g). In healthy controls and FTLD patients, CSF HMGB1 was unchanged, whereas a portion of the AD patients showed an increase in CSF HMGB1 up to 0.62 ng/ml. Interestingly, HMGB1 was also increased in several of the ALS patients who were part of the disease control group (Fig. 4g). These patients showed clinically typical AD and ALS. Interestingly, the patient with the highest levels of HMGB1 in the CSF (AD05) showed a rapid progression of dementia. At the initial consultation, within 5 years of possible onset, the MMSE score was 4, FAB was 6, and RCPM was impossible. Another AD patient (AD01) also showed a relatively rapid progression of dementia. Therefore, the results suggest that CSF HMGB1 could be a marker of progression of neurodegeneration regardless of the type of neurodegenerative disease. However, further investigation with a larger number of patients is essential. Considering the molecular weight of HMGB1 (29 kDa), which is far above the molecular weight cut-off of the blood-brain barrier (400–600 Da), the amount of HMGB1 in the blood may be lower. Additionally, technical development of an assay system will be needed for easier detection.

### Antibody therapy against HMGB1 ameliorates pathology and cognitive function in an AD mouse model

**Figure 5:**
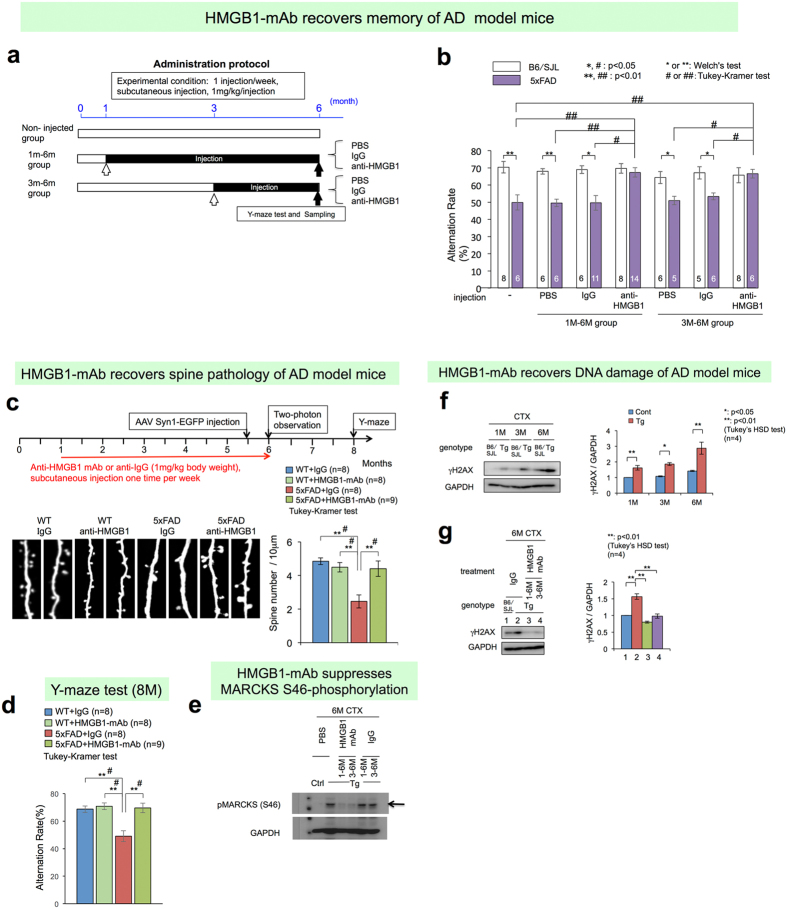
Anti-HMGB1 monoclonal antibody ameliorates symptoms and pathology in an AD mouse model. **(a)** Therapeutic protocol of 5xFAD mice subcutaneously injected with a monoclonal antibody against HMGB1. Mice were divided into a non-injected group, an injected group with PBS and an injected group with anti-HMGB1-monoclonal antibody (HMGB1-mAb). Injection protocols were further divided to 1–6 months and 3–6 months of age. The alteration rate in the Y-maze, the only method to detect memory disturbances in the early stage, was determined at 6 months of age based on a previous report^17^. **(b)** In the groups injected with HMGB1-mAb (both 1–6 and 3–6 months), the decrease in the alteration rate returned to normal levels, whereas the injection of PBS or IgG was ineffective. **(c)** Two-photon microscopic analysis of dendritic spines of an independent 5xFAD mouse group revealed that subcutaneous injection of HMGB1-mAb from 1 to 6 months recovered spine density at 6 months of age. **(d)** The mice used for two-photon microscopic analysis were bred for another 8 weeks and tested by the Y-maze at 8 months (32 weeks). Memory disturbances of the 5xFAD mice were still ameliorated. **(e)** MARCKS phosphorylation at Ser46 was tested in the cerebral cortex tissues of the mice used in Fig. 5B. HMGB1-mAb rescued the abnormal phosphorylation at Ser46. **(f)** Western blot of a DNA damage marker, γH2AX, revealed that DNA damage accumulates during ageing, especially in 5xFAD mice. **(g)** Subcutaneous injection of the HMGB1-mAb during 1–6 months or 3–6 months completely recovered DNA damage (γH2AX) in the cerebral cortex of 5xFAD mice at 6 months of age.

Finally, we tested whether an anti-HMGB1 antibody could ameliorate the symptoms and phenotypes of AD in a mouse model. We generated multiple monoclonal antibodies against HMGB1 and selected a clone, 2C8C, based on binding assays (Supplementary Figure 12). We subcutaneously injected the anti-HMGB1 monoclonal antibody into 5xFAD mice using two types of protocols (Fig. 5a). We confirmed delivery of the biotin-labelled IgG using a similar method and found that the concentration in the plasma and brain tissue increased to a level detected by ELISA (Supplementary Figure 13a) but not by immunohistochemistry (Supplementary Figure 13b). The Y-maze test sensitively detected the onset of 5xFAD at 6 months of age as previously reported^17^, and two protocols of subcutaneous injection of the monoclonal anti-HMGB1 antibody completely rescued the cognitive impairment of 5xFAD mice to the level of wild type mice (Fig. 5b).

Consistent with the results of the Y-maze test, an independent series of subcutaneous injections with the same antibody revealed the effect of the antibody on spine morphology by two-photon microscopy (Fig. 5c). Again, we confirmed the recovery of cognitive impairment by the Y-maze test in the new series of experiments (Fig. 5d). In addition, we confirmed that the anti-HMGB1 monoclonal antibody improved two other parameters. First, the injection of the anti-HMGB1 monoclonal antibody clearly reduced the number, area and intensity of the pSer46-MARCKS dots in the quantitative analyses (Supplementary Figure 14a,b). Western blot analysis supported the decrease of pSer46-MARCKS in the cortex of 5xFAD mice at 6 months of age (Fig. 5e). Second, the antibody reduced DNA damage in the cortex (Fig. 5f,g). DNA damage was increased during ageing in normal mice, and the extent of the increase was higher in the AD mice at all ages (Fig. 5f). Injection of the anti-HMGB1 antibody during 1–6 and 3–6 months of age clearly reduced the DNA damage in the cerebral cortex of 5xFAD mice to normal levels at 6 months (Fig. 5g).

However, the effect of the anti-HMGB1 monoclonal antibody on the number, area or intensity of the Aβ plaques was minimal (Supplementary Figure 14a,b). Although immunohistochemistry showed almost complete suppression of pSer46-MARCKS, the Aβ plaques were not changed remarkably (Supplementary Figure 14a). DAB staining showed that the number and intensity of Aβ plaques were slightly reduced (Supplementary Figure 14b). In addition, the area of Aβ plaques had also a tendency to decrease (Supplementary Figure 14B). However, western blotting with the same antibody (82E1) revealed that the total amount of all Aβ species^50^,^51^ was decreased in the brains of 5xFAD mice following injection of the anti-HMGB1 monoclonal antibody (Supplementary Figure 14c). In addition, the antibody appeared to reduce the amount of Aβ aggregates as well as the oligomers and ADDLs/protofibrils (Supplementary Figure 14c). The relationship between DNA damage (γH2AX) and neurite degeneration (pSer46-MARCKS) was reconfirmed (Supplementary Figure 14d). Western blot and qPCR analyses confirmed that the anti-HMGB1 monoclonal antibody did not affect expression of human APP in the brains of 5xFAD mice (Supplementary Figure 14e).

### Bi-directional suppression of polymerization between HMGB1 and Aβ

We investigated whether the effects of the anti-HMGB1 monoclonal antibody were mediated by a direct effect on HMGB1 toxicity or by a combination involving indirect effects on the Aβ polymerization process. An interaction between Aβ and HMGB1 has been reported^52^,^53^, but critical points remain uncertain or controversial, such as the effect of HMGB1 on the Aβ polymerization process, the effect on the Aβ monomer/oligomer/polymer ratio, and the effect of Aβ on HMGB1. Therefore, we examined these issues (Supplementary Figures 15 and 16).

First, we determined the conditions for testing the effect of HMGB1 on the aggregation process of the Aβ^1–^_42_ peptide (Supplementary Figure 15). We found that incubation for 48 hours at 37 °C was appropriate for observing aggregation by Western blot and electron microscopy, which is generally consistent with previous reports^54^,^55^. Next, we examined how HMGB1 affected the Aβ aggregation process in these conditions (Supplementary Figure 16). We assessed various Aβ species, such as monomers, oligomers (2–4-mer), ADDLs/protofibrils and fibrils^50^,^51^, and Western blot analysis revealed that HMGB1 clearly suppressed Aβ polymerization and increased the ratios of oligomer and ADDLs among all Aβ species (Supplementary Figure 16a). As expected, the addition of an anti-HMGB1 monoclonal antibody to the samples suppressed the increase of Aβ oligomers/ADDLs by HMGB1 but enhanced Aβ aggregates (Supplementary Figure 16a). These effects were not observed with a control IgG (Supplementary Figure 16a). Quantitative analysis supported these findings (Supplementary Figure 16b). Focusing on species other than fibrils/aggregates, we repeated the experiments and confirmed that the anti-HMGB1 monoclonal antibody suppressed the HMGB1-induced increase in Aβ monomers and oligomers/ADDLs (Supplementary Figure 16c).

We also noticed that HMGB1 polymerizes *in vitro*, and the addition of Aβ inhibited HMGB1 polymerization (Supplementary Figure 16d). Moreover, we found that the mixture of Aβ and HMGB1 produced three extra bands, possibly corresponding to heterocomplexes of Aβ and HMGB1 (Supplementary Figure 16d). Based on the molecular weight, these complexes were expected to be H1A1 (HMGB1: Aβ = 1:1) heterodimers, H1A2 heterotrimers and H2A2 heterotetramers (Supplementary Figure 16d). A commercial polyclonal anti-HMGB1 antibody recognized these Aβ-HMGB1 heterocomplexes, and the Western blot revealed that our anti-HMGB1 monoclonal antibody affected the formation of these heterocomplexes. The HMGB1 monomer (H1) increased and H2A2 decreased by addition of the anti-HMGB1 monoclonal antibody (Supplementary Figure 16d). The effect of the anti-HMGB1 monoclonal antibody on pure HMGB1 polymerization was also investigated, and we found that the monoclonal antibody inhibited polymerization of HMGB1 and increased the HMGB1 monomer (Supplementary Figure 16e). It is of note that an extremely high molecular weight substance reminiscent of fibrils was not found in the pure incubation of HMGB1.

The effect of HMGB1 on Aβ polymerization was also examined by electron microscopy (Supplementary Figure 16f). We found that HMGB1 inhibited the formation of Aβ fibrils, and the addition of the anti-HMGB1 monoclonal antibody recovered the formation of Aβ fibrils (Supplementary Figure 16e, black arrows). Collectively, these results indicate that Aβ and HMGB1 mutually inhibit polymerization of the other molecule. Consequently, HMGB1 inhibited fibril formation of Aβ and increased Aβ oligomers and protofibrils (Supplementary Figure 16a). The anti-HMGB1 monoclonal antibody inhibited polymerization of both Aβ and HMGB1, and in the case of Aβ polymerization, it reduced the Aβ oligomers and protofibrils in the presence of HMGB1 (Supplementary Figure 16a–c). Western blots did not show fibrils of HMGB1 (data not shown).

### Enhanced microglial phagocytosis by anti-HMGB1 monoclonal antibody

The enhanced fibril formation of Aβ *in vitro* by the anti-HMGB1 monoclonal antibody (Supplementary Figure 16a,b) was not consistent with the decrease in all Aβ species *in vivo* by the same antibody (Supplementary Figure 14a–c). Given that the total amount of all Aβ species was decreased in the brain after injection of the anti-HMGB1 monoclonal antibody (Supplementary Figure 14c), specific mechanisms related to the clearance of Aβ aggregates by an anti-Aβ antibody^56^ or other mechanisms, such as enhanced activity of phagocytosis, might exist. Interestingly, it was reported that TLR signalling is essential for the clearance of Aβ deposition by microglia^47^. Consistent with this idea, the anti-HMGB1 monoclonal antibody that blocks the ligand-receptor interaction might influence the TLR-stimulated phagocytosis of microglia. Another report suggested that the Aβ-HMGB1-complex was more easily phagocytosed by microglia^53^. Therefore, the anti-HMGB1 monoclonal antibody might suppress or even enhance Aβ phagocytosis by microglia.

Immunohistochemistry of a microglia-specific marker, Iba1, revealed that the number of microglia was increased by injection of anti-HMGB1 monoclonal antibody, especially around Aβ aggregates, and these microglia incorporated Aβ into the cytoplasm (Supplementary Figure 17a,b), supporting the hypothesis that the anti-HMGB1 monoclonal antibody enhances phagocytosis of the Aβ-HMGB1 complex. In addition, microglia phagocytosis of Aβ+ HMGB1+ anti-HMGB1 monoclonal antibody was higher than that of Aβ or of Aβ+ HMGB1 and in a microglial primary culture (Supplementary Figure 17c). These results suggest that the decreased Aβ deposition by the anti-HMGB1 monoclonal antibody was not due to the direct effect of HMGB1 on Aβ polymerization but resulted from enhanced Aβ phagocytosis of the Aβ/HMGB1/anti-HMGB1 monoclonal antibody complex by microglia. Presumably, this occurred through Fc receptor-mediated phagocytosis, as shown in the case of the anti-Aβ antibody^57^.

Finally, to understand the genetic interaction between Aβ and HMGB1, we tested whether extracellular Aβ could induce the release of HMGB1 from neurons. Thus, we quantified the release of HMGB1 into the medium of primary cultures of mouse cortical neurons after stimulation with three types of Aβ species by ELISA (Supplementary Figure 18). The results revealed that the Aβ monomer and oligomer could induce HMGB1 release from primary cortical neurons only when the Aβ species were present in the extracellular space at high concentrations of more than 1 μM. The activity of the Aβ fibril was weaker, and induction was not confirmed even at 1 μM (Supplementary Figure 18). Therefore, although it is theoretically possible, it is rare for extracellular Aβ to induce the release of HMGB1 from cells.

## Discussion

This study elucidated the role of HMGB1 in the pathology of AD, demonstrating that it is partially independent of yet closely related with Aβ. HMGB1 induces neurite degeneration independent of Aβ but also modifies the aggregation processes of Aβ and alters the balance among different Aβ species. The release of HMGB1 from necrotic neurons with intracellular Aβ (Fig. 2b,c)^48^ suggested that HMGB1 is downstream of the intracellular toxicity of Aβ. However, as shown in the last experiment (Supplementary Figure 18), the extracellular toxicity of Aβ was not considered a plausible mechanism. Given that HMGB1 is released from hyperexcitatory neurons irrespective of Aβ, and HMGB1 clearly modifies the polymerization of Aβ and increases Aβ oligomers, HMGB1 can also be considered to be located upstream of Aβ. Taken together, the genetic interaction between Aβ and HMGB1 is bi-directional. In this regard, HMGB1 might be considered an independent mediator of AD that has a close relationship with the amyloid cascade.

The direct pathway from Aβ to neurite degeneration via MARCKS phosphorylation at Ser46 was assessed. However, because the necessary concentration for Aβ to trigger phosphorylation was much higher than that of HMGB1 (Supplementary Figure 11d) and because Aβ was more prone to aggregate in the extracellular space than HMGB1 (Supplementary Figure 9a,b), HMGB1 may be the primary trigger of the pathological signalling via pSer46-MARCKS in AD. We also presented data showing that HMGB1 was released from living neurons under hyperexcitation (Fig. 4a,b). However, as described in the text, we determined that the hyperexcitation-induced increase in HMGB1 concentration in the extracellular space was insufficient to trigger phosphorylation of MARCKS at Ser46. Further investigation is necessary to address whether chronic hyperexcitation in the default mode network or other brain areas triggers the phosphorylation of MARCKS at Ser46.

This study also provides a new tool to modify the progression of AD by targeting HMGB1. Our results suggest three possible mechanisms for how the anti-HMGB1 monoclonal antibody ameliorates AD pathology (Supplementary Figure 19). First, the anti-HMGB1 monoclonal antibody blocks HMGB1 activity through TLR4, suppresses phosphorylation of MARCKS at Ser46, and prevents neurite degeneration as well as dendritic spine dysfunction. Second, the anti-HMGB1 monoclonal antibody suppressed the formation of the Aβ oligomer, which is more toxic than the HMGB1 monomer, through modification of an interaction between Aβ and HMGB1 in the extracellular space. Third, the anti-HMGB1 monoclonal antibody increased phagocytosis of all Aβ species by microglia, presumably through an interaction with Aβ-HMGB1 heteromers. Given that the anti-HMGB1 monoclonal antibody suppressed neurite degeneration even after the formation of Aβ aggregates in the brain (treatment from 3 to 6 months), the activity of the anti-HMGB1 monoclonal antibody appeared to be mainly due to the independent function of HMGB1 (the first mechanism), although our results do not exclude the possibility that the antibody also suppresses the toxicity of Aβ oligomers or aggregates (the second and third mechanisms).

In conclusion, this study revealed that HMGB1 acts as a mediator of neurite degeneration through the identification of the pathological signalling pathway in AD. The signalling pathway is initiated with the extracellular release of HMGB1, followed by activation of the TLR4-MAP kinases, phosphorylation of MARCKS at Ser46, and neurite degeneration via instability of the actin network. This study also revealed that preclinical antibody therapy targeting neurite degeneration (immunotherapy with anti-HMGB1 monoclonal antibody) is effective in delaying the onset of disease even after aggregation of Aβ.

## Materials and Methods

### Mice

5xFAD transgenic mice overexpressing the mutant human APP (770) with the Swedish (KM670/671NL), Florida (I716V), and London (V717I) familial Alzheimer’s disease (FAD) mutations and human PS1 with FAD mutations (M146L and L285V) were purchased from The Jackson Laboratory (CA, USA). Both the APP and PS1 transgenes were under the control of the mouse *Thy1* promoter^17^. The backgrounds of the mice were C57BL/SJL, which was produced by crossbreeding C57BL/6J female and SJL/J male mice. Using 5xFAD mice, we performed a comprehensive proteomics analysis with brain tissues of male mice at 1, 3, 6 and 12 months of age as previously described^9^. APP-KI mice, which possesses a single human APP gene with Swedish (KM670/671NL) and Beyreuther/Iberian (I716F) mutations, was described previously^22^.

### Human brain samples

For the proteome analysis, temporal and occipital pole brain samples were dissected from five AD and five control patients and deep-frozen (−80 °C) within 1 hour of death. The pathological diagnosis of the brains was established based on immunohistochemistry findings, and only pure AD cases without other pathological changes, such as Lewy bodies, TDP-43 cytoplasmic aggregates, or argyrophilic grains, were used. Control brains were derived from age-matched patients who died due to non-neurological diseases.

### Phosphoproteome analysis

Phosphorylated proteins were prepared from mouse and human cerebral cortices as previously described^9^. Briefly, brain extracts were denatured by detergent and heat treatment and then reduced to block the cysteine bond. The protein extracts were digested with trypsin. The deduced phosphopeptides were enriched using the Titansphere Phos-TiO Kit (GL Sciences Inc., Japan), labelled using an iTRAQ Reagent multiplex kit (SCIEX Ins.) and subjected to strong cation exchange (SCX) chromatography. Each fraction was analysed using a DiNa Nano-Flow LC system (KYA Technologies Corporation, Japan) and 0.1 mm × 100 mm C18 columns (KYA Technologies Corporation, Japan). The ion spray voltage applied to the sample from the Nano-LC to the Triple TOF 5600 System (SCIEX Ins.) was 2.3 kV. The information-dependent acquisition (IDA) setting was 400–1250 *m/z*. Acquisition and analysis of mass spectrum data of peptides were performed by Analyst TF (version 1.6) (SCIEX Ins.). Using a Paragon algorithm^58^, ProteinPilot (version 4.5) (SCIEX Ins.) was used as the database to search for corresponding mouse and human proteins of identical masses, and the identified proteins were grouped by the ProGroup algorithm (SCIEX Ins.) to exclude redundancy. Only the proteins with more than 95% confidence in identification were accepted and used for further analysis in this study. Phosphopeptide ratios, the ratio of peptide quantities of AD samples compared to those of control samples, were calculated from reporter signals of the two groups that had a greater than 95% confidence. Phosphorylated sites were identified in reference to the UniProt database. Changes in phosphopeptides of the AD samples were calculated as the ratios to the control samples. Phosphopeptide ratios were considered to follow log-normal distribution, and P-values were calculated by a two-tailed Welch’s test using the log ratio. Changes in phosphopeptides were judged significant at P < 0.05.

### Western blot analysis

Mouse cerebral cortex tissues or primary cortical neurons were homogenized with a plastic homogenizer (Bio-Masher II, Nippi, Tokyo, Japan) after the addition of lysis buffer [100 mM Tris-HCl (pH 7.5, Sigma, MO, USA), 2% SDS (Sigma, MO, USA), 1 mM DTT (Sigma, MO, USA) and a protease inhibitor cocktail (Calbiochem, #539134, 1:200 dilution)]. Lysates were incubated in a rotator for 30 min at 4 °C and then boiled at 100 °C for 15 min. After centrifugation (16,000 × *g* × 10 min at 4 °C), the supernatants were diluted with an equal volume of sample buffer [125 mM Tris-HCl (pH 6.8, Sigma, MO, USA), 4% SDS (Sigma, MO, USA), 20% glycerol (Wako, Osaka, Japan), 12% mercaptoethanol (Wako, Osaka, Japan), and 0.05% BPB (Nacalai, Kyoto, Japan)]. These samples were separated by SDS-PAGE, transferred to Immobilon-P polyvinylidene difluoride membranes (Millipore, MA, USA) through a semi-dry method, and blocked by 2% BSA (Nacalai, Kyoto, Japan) or 5% milk in TBST (10 mM Tris-HCl, pH 8.0, 150 mM NaCl, 0.05% Tween-20). Primary and secondary antibodies were diluted in TBST with 0.2% BSA or Can Get Signal solution (Toyobo, Osaka, Japan) as follows: mouse anti-actin, 1:1000 (sc-47778, Santa Cruz Biotechnology, TX, USA); rabbit anti-GFP, 1:1000 (sc-8334, Santa Cruz Biotechnology, TX, USA); rabbit anti-phospho-MARCKS (Ser46), 1:100,000 [ordered from GL Biochem (Shanghai) Ltd., Shanghai, China]; mouse anti-MARCKS, 1:1000 (sc-100777, Santa Cruz Biotechnology, TX, USA); mouse anti-GAPDH, 1:3000 (MAB374, Millipore, MA, USA); rabbit anti-TLR4, 1:1000 (NB100-56566, Novus, MI, USA); mouse anti-amyloid β, 1:5000 (clone 82E1, IBL, Gumma, Japan); mouse anti-phosphorylated H2AX (γH2AX), 1:3000 (Millipore, JBW301, MA, USA); HRP-linked anti-rabbit IgG, 1:3000 (NA934, GE Healthcare, Buckinghamshire, United Kingdom); and HRP-linked anti-mouse IgG, 1:3000 (NA931, GE Healthcare, Buckinghamshire, United Kingdom). Primary and secondary antibodies were incubated overnight at 4 °C and for one hour at room temperature, respectively. ECL Prime Western Blotting Detection Reagent (RPN2232, GE Healthcare, Buckinghamshire, United Kingdom) and a luminescent image analyser (ImageQuant LAS 500, GE Healthcare, Buckinghamshire, United Kingdom) were used to detect proteins.

### Immunohistochemistry

For immunohistochemistry, mouse or human brains were fixed with 4% paraformaldehyde and embedded in paraffin. Sagittal or coronal sections (5 μm thickness) were obtained using a microtome (Yamato Kohki Industrial Co., Ltd., Saitama, Japan). Immunohistochemistry was performed using primary antibodies as follows: rabbit anti-phospho-MARCKS (Ser46), 1:2,000 [ordered from GL Biochem (Shanghai) Ltd., Shanghai, China]; mouse anti-amyloid β, 1:1000 (clone 82E1, #10323, IBL, Gumma, Japan); mouse anti-MAP2, 1:200 (sc-32791, Santa Cruz Biotechnology, TX, USA); rabbit anti-HMGB1, 1:200 (ab18256, Abcam, Cambridge, UK); and mouse anti-tau, 1:2000 (MA5-15108, Thermo Fisher, MA, USA). Reaction products were visualized with Alexa Fluor-488- or -568-conjugated secondary antibodies (Molecular Probes, MA, USA). Amyloid β was additionally visualized with a Vectastain Elite ABC kit (PK-6100, Vector Laboratories, USA) and a DAB Peroxidase Substrate kit (SK-4100, Vector Laboratories, USA). Nuclei were stained with DAPI (0.2 μg/ml in PBS, #D523, DOJINDO Laboratories, Kumamoto, Japan). All images were acquired using fluorescence microscopy (Olympus IX70, Tokyo, Japan), light microscopy (Olympus, Tokyo, Japan) or confocal microscopy (Olympus FV1200IX83, Tokyo, Japan).

### Three-dimensional structure imaging by confocal microscopy

Co-staining of Aβ/pMARCKS (Ser46)/DAPI; pMARCKS (Ser46)/MAP2/DAPI; pMARCKS (Ser46)/tau/DAPI; or amyloid β/HMGB1/DAPI was acquired with 0.5 μm Z-stacks at 40x magnification on an Olympus FV1200IX83 (Olympus, Tokyo, Japan). Multiple slice information was converted to 3D images using IMARIS 7.2.2 (Bitplane, Switzerland), and a 3D surface model was generated as the final image with further image processing (Image Smoothing and Image Thresholding mode).

### Generation of hybridoma cell clones for an anti-HMGB1 monoclonal antibody

Full-length HMGB1 cDNA from Wistar rats was subcloned into the pGEX-3X vector (GE Healthcare, Buckinghamshire, England). The protein sequences of Wistar rats and mice are identical, whereas rat and human HMGB1 are identical except for two residues located in the C-terminal positively/negatively charged region (Supplementary Figure 11c). HMGB1-GST protein (100 μg) was mixed with TiterMax® Research Adjuvant (CytRx Corporation, LA, USA) and injected into the footpad of BALB/c female mice (5 weeks of age) every two days for four times total. After another two days, lymphocytes were isolated and fused to mouse myeloma cells by the PEG method. Fused cells were cultured in 96-well-plates in the selection medium containing FCS, aminopterin, streptomycin, and penicillin for 10 days at 37 °C in 5% CO_2_. Aliquots of the medium from 96-well-plates were tested by ELISA for the production of anti-HMGB1 monoclonal antibodies. In brief, 250 ng of HMGB1 protein and a negative control protein (GST) were fixed to the bottom of microplate wells, and aliquots of the medium were added. After incubation for 1 hour at 37 °C, the microplates were washed with PBS three times, incubated with the secondary antibody [HRP-labelled anti-mouse IgG (MBL, Cat. #330, Nagoya, Japan)] for 1 hour at room temperature, washed another three times with PBS, incubated with TMB Ultra Sensitive Substrate (MOSS, Inc., Chicago, IL, USA, USA), and the absorbance was read at 450 nm (reference at 550 nm) in a plate reader.

### Antibody purification

The selected hybridoma cell clones were incubated in serum-free medium, Hybridoma-SFM (Thermo Fisher Scientific, MA, USA), and the medium from the expanded culture of the selected clones was centrifuged to exclude cell debris. Monoclonal antibodies in the semi-purified medium bound to rProtein A Sepharose Fast Flow resin (GE Healthcare, Buckinghamshire, England) overnight using a circulation pump. Then, the columns were washed with 10x the volume of PBS, and the bound monoclonal antibodies were eluted with 0.1 M sodium citrate (pH4.0), neutralized with 1.0 M Tris-HCl (pH 9.0), and subjected to dialysis with PBS using the recommended protocol.

### Subcutaneous injection of anti-HMGB1 monoclonal antibody

5xFAD or B6/SJL mice received subcutaneous injections of 1 mg/kg of control IgG (Protein A-purified mouse IgG2a antibody produced by a hybridoma cell line at MBL Co., Ltd.) or the anti-HMGB1 monoclonal antibody once a week in the dorsal neck region from 1 to 6 months of age or from 3 to 6 months of age.

### Transfer of biotin-labelled antibody to plasma and brain

Biotin-labelled mouse IgG (SAB3700901, Sigma-Aldrich, MO, USA) was injected subcutaneously in a similar manner to the anti-HMGB1 monoclonal antibody. Sampling of blood plasma and brain tissues was performed at day 1 and day 3.

To detect the transfer to plasma and brain tissues, a sandwich ELISA system was generated as follows. Anti-mouse IgG (50 μl, 10 μg/ml, Cat.115-005-071, Jackson ImmunoResearch, PA, USA) in PBS was added to each well of the plates and left overnight at 4 °C. After washing with PBS, 400 μl of 3% BSA and 400 μl of 0.05% Tween-20 in PBS was added to each well and incubated for 2 hours at room temperature for fixation. As a negative control, anti-rabbit mouse IgG (Sigma-Aldrich, SAB3700901, MO, USA) was similarly fixed to the bottom of the wells. After washing with 0.1% Tween-20 in PBS and 3% BSA, the brain tissues or blood samples sequentially diluted with 0.05% Tween-20 in PBS were added to the wells and reacted with the fixed anti-mouse IgG for 2 hours at room temperature. During the incubation, the reaction solution was made as follows. A drop of solution A and B (PK-6100 VECTASTAIN Elite ABC Standard Kit, Vector Laboratories, Burlingame, CA, USA) was added to 3% BSA and 0.05% Tween-20 in 2.5 ml of PBS and rotated for 30 min. After washing the wells three times with 0.1% Tween-20 in PBS, 50 μl of the reaction solution was added to the well and reacted for 45 min at room temperature. Then, the wells were washed three times with 0.1% Tween-20 in PBS. The TMB-1-component Sure Blue (50 μl, Kirkegaard & Perry Laboratories, Gaithersburg, MD, USA) was added to start the reaction and incubated for 15 min, and the reaction was stopped with 1 N HCl. Absorbance was measured using a plate reader (Multiscan Ascent, Thermo Labsystems, Helsinki, Finland) at 450 nm.

### Y-maze test

Exploratory behaviour was performed in a Y-shaped maze consisting of three identical arms with equal angles between each arm (O’HARA & Co., Ltd, Tokyo, Japan). Mice at the age of 6 months were placed at the end of one arm and allowed to move freely through the maze during an 8 min session. The percentage of spontaneous alterations (indicated as an alteration rate) was calculated by dividing the number of entries into a new arm that was different from the previous one with the total number of transfers from an arm to another arm.

### Generation of different Aβ species

Aβ oligomers, protofibrils/ADDLs and aggregates/fibrils were prepared according to the method previously described^59^. In brief, lyophilized amyloid beta 1–42 (Aβ_1–42_) protein (Peptide Institute, Osaka, Japan) was dissolved in DMSO (Sigma, MO, USA) as a 1 mM stock solution. The Aβ_1–42_ stock solution was diluted to 100 μM for cell cultures or 30 μM for *in vitro* analysis with PBS. For oligomer and ADDL formation, an additional incubation was performed at 5 °C for 24 hours and for aggregate/fibril formation at 37 °C for 24 hours.

### Tris-Tricine SDS-PAGE

Aβ formation was tested by Tris-Tricine SDS-PAGE as previously described^60^. In brief, the electrophoresis gel consisted of 0.1% SDS, 0.1 g/ml of glycerol, and 0.1 M Tris and was adjusted to pH 8.45 by HCl. Aβ samples were separated by Tris-Tricine SDS-PAGE using an anode buffer with 1 M Tris-Cl (pH 8.9) and a cathode buffer with 0.1 M Tris and 0.1 M Tricine. The 16, 10 and 4% gels were layered from bottom to top.

### Transmission electron microscopy

Each sample from the *in vitro* aggregation formation assay was fixed with 2% glutaraldehyde and placed on a 300-mesh copper grid that had been coated with formvar for 1 min, washed with water, and negatively stained with 1% uranyl acetate for 1 min. Samples were observed with a transmission electron microscope (HITACHI, H-7100, Tokyo, Japan) operating at 80 kV.

### Immunoelectron microscopy

Each sample from the *in vitro* aggregation formation assays was placed on a nickel grid and fixed with 1% PFA in 0.1 M phosphate buffer for 10 min at room temperature, washed with 0.1 M Tris-HCl, pH 7.5, and incubated with 5% goat serum in 0.1 M Tris-HCl, pH 7.5 for 10 min to exclude non-specific binding of the antibody. It was then incubated with primary antibody [mouse anti-Aβ (diluted at 1:50, clone 6E10, SIG-39300, Covance, NJ, USA) or rabbit anti-HMGB1 (diluted at 1:50, ab18256, Abcam, Cambridge, UK)] in 5% goat serum in 0.1 M Tris-HCl pH 7.5 for 2 hours at room temperature, washed with 0.1 M Tris-HCl pH 7.5, and incubated with secondary antibody [anti-mouse 10 nm gold-conjugated (diluted at 1:100, EM GAF 10, BB International, Cardiff, UK) or anti-rabbit 5 nm gold-conjugated (diluted at 1:100, EM GAR 5, BB International, Cardiff, UK)] for 1 hour at room temperature with 0.1 M Tris-HCl pH 7.5. The samples were negatively stained with 1% uranyl acetate for 1 min and used for TEM observation.

### HMGB1 ELISA with human cerebrospinal fluid

Cerebrospinal fluid (CSF) samples were obtained from non-neurological patients diagnosed after various laboratory or X-ray examinations or AD, FTD and ALS patients. All samples were stored at −80 °C until use. The CSF was analysed for HMGB1 levels using an HMGB1 ELISA Kit II (Shino-Test Corporation, Tokyo, Japan) following the manufacturer’s instructions. Briefly, 40 μl of CSF sample was diluted up to 110 μl with dilution buffer and incubated in an ELISA plate coated with anti- HMGB1 antibody for 24 hours at 37 °C. The plates were washed, and 100 μl of HRP-conjugated antibody was added and incubated for 2 hours at room temperature (RT). The wells were washed, and 100 μl of substrate solution was added to each well and incubated for 30 min at room temperature. Finally, 100 μl of stop solution was added to each well, and the absorbance was measured at 450 nm using a plate reader.

### Photo-stimulation of primary cortical neurons

Primary cortical neurons obtained from C57BL/6 J mouse embryos (E15) were cultured in 24-well plates at densities of 3.5 × 10^5^ cells per well in the presence of AraC to eliminate glial cells. The neurons were infected with AAV-CAG-ChR2-EGFP (#26929, Addgene, MA, USA) at an MOI of 5 on day 3. Photo-stimulation with 470 nm LEDs with a power of 34 W was carried out 2 weeks after infection. Light (1000 lumens) was cast in a 63 cm^2^ area, and the final luminance was approximately 1.5 Mlx. The protocol of stimulation programmes is shown in Fig. 4A. Medium was replaced with B27 supplement (Gibco, Thermo Fisher Scientific, Waltham, MA, USA) -free neurobasal medium (Gibco, Thermo Fisher Scientific, Waltham, MA, USA) before starting photo-stimulation to prevent contamination of HMGB1, which is included in the B27 supplement.

### *In vitro* phosphorylation of MARCKS by candidate kinases

To generate the MARCKS protein as the substrate, we constructed a GST-fusion protein of human truncated MARCKS (1–176). Human MARCKS (1–176) cDNA was amplified from the pEGFP-C1-human MARCKS (wild type) vector using primers (5′-ATGCGAATTCATGGGTGCCCAGTTCTCCA-3′ and 5′-ATGCCTCGAGTTACTTCTTGTTCTTCTTGAAGGAG-3′) containing *EcoRI* and *XhoI* sites and subcloned into pGEX-6P-1 (GE Healthcare, Buckinghamshire, United Kingdom). The pGEX-6P-1-human MARCKS (1–176) plasmid was transformed into *E. coli* Rosetta (DE3) (Novagen) competent cells, which were cultured in LB broth in a shaker at 200 rpm and 37 °C until the OD_600_ reached 0.3–0.4. After addition of IPTG (final concentration 1.0 mM), the culture was incubated for another 2 hours. The *E. coli* cells were collected by centrifugation and lysed in PBS containing 0.1% Tween-20, 0.1% lysozyme (Sigma, MO, USA), and 1/500 volume of protease inhibitor cocktail III–EDTA-free (Calbiochem). The extract was sonicated seven times for 15 sec in 1 minute intervals using output level 6 (ultrasonic homogenizer UH-50, SMT Company, Japan) and centrifuged at 15,000 × *g* for 20 min at 4 °C. The supernatant was added to Glutathione Sepharose 4B resin (GE Healthcare, Buckinghamshire, United Kingdom) equilibrated with PBS containing 0.1% Tween-20 and rotated slowly for 3 hours at 4 °C. The suspension was applied to an empty column at 4 °C. The Glutathione Sepharose 4B column was washed with PBS containing 0.1% Tween-20 and eluted with 10 mM glutathione in PBS containing 0.1% Tween-20 at 4 °C by gravity flow. The GST-human MARCKS (1–176) fusion protein fractions were desalted with PD-10 columns (GE) using PBS containing 0.01% Tween-20. Human MARCKS (1–176) protein (30 pmol) and 1.5 pmol of human ERK1, ERK2 (SignalChem Inc.), JNK1, JNK2 or JNK3 (Carna Biosciences, Inc.) were mixed with 30 μl of kinase buffer [25 mM Tris-HCl (pH 7.5), 2 mM DTT, 5 mM β-glycerophosphate, 0.1 mM Na_3_VO_4_, 10 mM MgCl_2_, 200 μM ATP] for 2 hours at 37 °C.

### SWATH-Mass analysis of *in vitro* phosphorylation products

Samples from the *in vitro* phosphorylation reaction were enriched with the Titansphere Phos-TiO Kit (GL Sciences Inc., Japan). After desalting, the samples were dried and dissolved in 35 μl of 0.1% formic acid. Aliquots of 5 μl were applied to a C18 column (0.1 mm × 100 mm, KYA Technologies Corporation, Japan) with solution A (0.1% formic acid), eluted with a gradient of 2–40% solution A and B (99.9% acetonitrile and 0.1% formic acid) using a flow rate of 300 nl/min in an Eksigent NanoLC-Ultra 1D Plus system (Sciex Ins.), and subjected to a TripleTOF 5600 system (Sciex) at 2.3 kV of ion spray voltage. The information-dependent acquisition (IDA) was set at 400–1000 *m/z* with two to five charges, and the product ion MS/MS scan range was between 100 and 1600 Da, with an accumulation time of 100 ms for a spectral library. SWATH (sequential window acquisition of all theoretical mass spectra) acquisition was performed by 24 sequential windows of 25 Da that spanned from 400 to 1000 Da. SWATH acquisition of the MS/MS spectral data^58^ was performed by the Analyst TF1.6 software (Sciex Ins.) for 100 ms/window, and the MS/MS spectral library was prepared by the Protein Pilot software (version 4.5). Peakview software (version 1.2.0.3, Sciex Ins.) correlated the MS/MS spectral data with peptide data and LC retention time. The MS/MS product ions from the same peptide were summed and used as the quantity of the peptide.

### Microglial phagocytosis of Aβ

Primary cortical microglia were prepared from Wistar rats (P0) as previously described^61^ and placed in eight-well chamber glass slides (Lab-Tek II, Nalgene, IL, USA) without coating at a density of 2 × 10^4^ cells/well. Pre-incubated TAMRA-Aβ (10 nM, #AK13A, Cosmo Bio Co. Ltd., Tokyo, Japan), TAMRA-Aβ/HMGB1 (10 nM each), or TAMRA-Aβ/HMGB1/anti-HMGB1-monoclonal antibody (10 nM each) was added to the primary cultures. After 45 min, the cells were fixed with 1% PFA for 20 min. Images were acquired by an Olympus FV1200-IX83 (Olympus, Tokyo, Japan). The percentage of TAMRA-Aβ-incorporated microglia was counted in 10 visual fields of 20x objective lens images, and the number of microglia incorporating TAMRA-Aβ was counted in each group.

### Three-dimensional imaging of transparent mouse brains

Transparent mouse brains were generated by the Sca*l*eS method as previously described^23^. In brief, Sca*l*eS solutions were made using urea crystals (Wako Pure Chemical Industries, 217–00615), D(−)-sorbitol (Wako Pure Chemical Industries, 199–14731), methyl-β-cyclodextrin (Tokyo Chemical Industry, M1356), γ-cyclodextrin (Wako Pure Chemical Industries, 037–10643), N-acetyl-L-hydroxyproline (Skin Essential Actives, Taiwan), dimethyl sulfoxide (DMSO) (Wako Pure Chemical Industries, 043-07216), glycerol (Sigma, G9012), and Triton X-100 (Nacalai Tesque, 35501-15). A mouse brain of a human mutant APP knock-in mouse^22^ was fixed at indicated ages and cleared with Sca*le*S. For immunohistochemistry, the following fluorophore-conjugated antibodies (Abs) were used: mouse monoclonal antibody to amyloid-β conjugated to Alexa Fluor-488 (Covance; SIG-39347, 1:200), rat Ab to pSer46-MARCKS conjugated to CF633 (Biotium) and goat synapsin-1 antibody conjugated to Oyster 550 (Luminartis). The images were acquired with a TPEFM system (Olympus FVMPE-RS) with 920-nm excitation and an objective lens (XLSLPLN25XGMP) with a numerical aperture (NA) = 1.0, working distance (WD) = 8 mm, refractive index (RI) = 1.41–1.52, a collection collar, and a z-drive. The *z* step was 2.0 μm.

### Quantitative PCR analysis of human APP expression

Total RNA was purified with NucleoSpin RNAII (MACHEREY-NAGEL GmbH & Co. KG, Düren, Germany). To eliminate genomic DNA contamination, on-column DNA digestion was carried out for each sample with DNase I. The purified total RNA was reverse-transcribed with SuperScript VILO (Invitrogen, Carlsbad, CA, USA). Quantitative PCR analyses were performed with the 7500 Real-Time PCR System (Applied Biosystems, Foster City, CA, USA) using the Thunderbird SYBR Green (TOYOBO, Osaka, Japan). The primer sequences were; mThy1_3F: CAGCAACTGGAGGCGTTGG for a mouse thy-1 promoter and hAPP_56R: TCCCACTCGCACAGCAGC for a 5′ untranslated region of human APP in a transgenes; rGAPDH_2L: 5′-AGCCCAGAACATCATCCCTG-3′ and rGAPDH_2R: 5′-CACCACCTTCTTGATGTCATC-3′ for mouse GAPDH.

### Statistics

For the phospho-proteome analysis, peptide ratios followed log-normal distribution. Therefore, the data are represented as the geometric mean ± standard error (s.e.m.), and the differences between AD samples and control samples were tested using the log ratio analysis. A two-tailed Welch’s test was applied to compare the differences in changes of phosphopeptides between AD samples and control samples. The significance level was set at 5%. For biological analyses, the data were considered to follow a normal distribution and are represented as the mean ± standard error. Student’s t-test was applied for two group comparisons. For multiple group comparisons, Tukey’s HSD test or Dunnett’s comparison were applied. The significance level was set at 1% or 5%. For Supplementary Figure 13, because the data did not show a normal distribution, the measured values were plotted directly. Non-parametric Wilcoxon’s rank-sum test was employed for this analysis. Differences were judged significant at a 5% significance level.

### Ethics for Animal experiments

This study was performed in strict accordance with the ARRIVE guidelines (Animal Research: Reporting *in vivo* Experiments) for the Care and Use of Laboratory Animals of the National Institutes of Health. It was approved by the Committees on Gene Recombination Experiments and Animal Experiments of Tokyo Medical and Dental University (Numbers: 2016-007A and 0170032A).

### Ethics for Human experiments

All experiments with human samples were performed after obtaining informed consent, and carried out in accordance with the approved guidelines for human experimental research. The experiments were approved by the Committee on Human Ethics of the Tokyo Medical and Dental University (Number: 2014-5-4).

## Additional Information

**How to cite this article**: Fujita, K. *et al*. HMGB1, a pathogenic molecule that induces neurite degeneration via TLR4-MARCKS, is a potential therapeutic target for Alzheimer's disease. *Sci. Rep.* **6**, 31895; doi: 10.1038/srep31895 (2016).

## Supplementary Material

Supplementary Information

Supplementary Video 1

Supplementary Video 2

Supplementary Video 3a

Supplementary Video 3b

Supplementary Video 3c

Supplementary Video 4
