# Supplementary material for: HMGB1, a pathogenic molecule that induces neurite degeneration via TLR4-MARCKS, is a potential therapeutic target for Alzheimer’s disease
Source: Sci Rep. 2016 Aug 25;6:31895. doi: 10.1038/srep31895 (PMC4997258; doi:10.1038/srep31895)

HMGB1, a pathogenic molecule that induces neurite degeneration via TLR4-MARCKS, is a potential therapeutic target for Alzheimer's disease

Kyota Fujita[1,$], Kazumi Motoki[1,$], Kazuhiko Tagawa[1,$], Xigui Chen[1], Hiroshi Hama[2], Kazuyuki Nakajima[3], Hidenori Homma[1], Takuya Tamura[1], Hirohisa Watanabe[4], Masahisa Katsuno[4], Chiemi Matsumi[5], Masunori Kajikawa[5], Takashi Saito[6], Takaomi Saido[6], Gen Sobue[4], Atsushi Miyawaki[2] and Hitoshi Okazawa[1,7,#]

1: Department of Neuropathology, Medical Research Institute, Tokyo Medical and Dental University, 1-5-45 Yushima, Bunkyo-ku, Tokyo 113-8510, Japan

2: Laboratory for Cell Function Dynamics, Brain Science Institute, RIKEN, 2-1 Hirosawa, Wako, Saitama 351-0198, Japan

3: Department of Bioinformatics, Institute of Bioinformatics, Soka university, 1-236 Tangi-machi, Hachioji, Tokyo 192-8577, Japan

4: Department of Neurology, Nagoya University Graduate School of Medicine, 65 Tsurumai-cho, Showa-ku, Nagoya 466-8550, Japan

5: Medical and Biological Laboratories Co., LTD. 4-5-3 Sakae, Naka-ku, Nagoya 460-0008, Japan

6: Laboratory for Proteolytic Neuroscience, Brain Science Institute, RIKEN, 2-1 Hirosawa, Wako, Saitama 351-0198, Japan

7: Center for Brain Integration Research, Tokyo Medical and Dental University, 1-5-45 Yushima, Bunkyo-ku, Tokyo 113-8510, Japan

$: KF, KM, KT are co-first authors

#: corresponding author

E-mail: okazawa-tky@umin.ac.jp

Supplementary Figure Legends

Supplementary Figure 1

Region-specific distribution of pSer46-MARCKS in 5xFAD mice

The distribution of Ser46-phosphorylation of MARCKS was examined in the brains of 5xFAD mice at 6 months of age (upper panels). Plaque-like stains of pSer46-MARCKS were found in 5xFAD mice but not in wild type mice (B6/SJL). Staining was not found in the cerebellum of 5xFAD mice, consistent with the cerebrum-dominant pathology of AD. Another antibody against pThr143-MARCKS did not show the plaque-like patterns (lower panels).

Supplementary Figure 2

Specificity of the anti-pSer46-MARCKS antibody

The specificity of anti-pSer46-MARCKS antibody was evaluated together with a commercial anti-MARCKS antibody (Santa Cruz Biotechnology) using lysates of HeLa cells expressing EGFP (negative control), MARCKS-EGFP (positive control) and EGFP-MARCKS (positive control). Both antibodies detected specific bands of MARCKS-EGFP (red arrow) and EGFP-MARCKS (blue arrow), whereas the two antibodies commonly reacted with non-specific bands (black arrows). One non-specific band was additionally detected by the anti-pSer46-MARCKS antibody (asterisk). Specific bands but not non-specific bands were absorbed by the phospho-MARCKS peptide used for generation of the antibody at 1:30 and 1:300 molar ratios between the phospho-MARCKS antibody and the phospho-MARCKS synthetic peptide.

Supplementary Figure 3

Relationship between pSer46-MARCKS staining and Aβ aggregation in the cerebral cortex of 5xFAD mice

(a) Images of co-stained brain tissues acquired by confocal fluorescence microscopy were further analysed using software (Imaris, Zeiss), and 3D images were generated. Three representative regions are shown. Blue and red enclosures indicate that the images are used for Figure 2B and enlarged images in Sup Figure 3 in the following.

(b) Two enlarged images from Sup Figure 3A. pSer46-MARCKS stains surrounded but not merged with Aβ aggregation.
(c) A representative image showing the connection between MAP2 and pSer46-MARCKS stains.

Supplementary Figure 4

Relationship between pSer46-MARCKS staining and Aβ aggregation in the cerebral cortex of PS1 mutant (M146L) human patients

(a) Images of the cerebral cortex of human PS1-linked AD patients were analysed similarly. Three representative regions are shown. The blue enclosure indicates that the images are used for Figure 2C.
(b) A representative image showing the connection between tau and pSer46-MARCKS staining.

Supplementary Figure 5

Relationship between pSer46-MARCKS staining and Aβ aggregation in the transparent brain of human APP knock-in mice

The forebrains of human mutant APP knock-in mice were made transparent by the Sca*l*eS method and subjected to immunohistochemistry of Aβ, pSer46-MARCKS and synapsin.

Supplementary Figure 6

Relationship between pSer46-MARCKS staining and two types of glia or neurons

(a) Images were obtained from the retrosplenial dysgranular cortex of 5xFAD mice co-stained with microglia- or astrocyte-specific markers. Phosphorylation of MARCKS at Ser46 did not occur in the microglia or astrocytes.
(b) Co-staining of NeuN, a neuronal marker, and pSer46-MARCKS revealed that phosphorylation of MARCKS at Ser46 occurred in the neurons, which were surrounded by pSer46-MARCKS-positive degenerative neurites. Groups 1, 2, and 3 are tentatively classified from the hypothetical progression. Group 1 is the first stage, Group 2 loses viability of neurons and reactivity of NeuN, and in Group 3

Supplementary Figure 7

Effect of Ser46-MARCKS phosphorylation on the interaction with actin

The interaction between MARCKS and actin affects dendritic spine morphology[14]. Phosphorylated mimics of full-length MARCKS-EGFP (S46D and S46E) lost the high affinity to actin, whereas a non-phosphorylated form of full-length MARCKS-EGFP (S46A) preserved the high affinity.

Supplementary Figure 8

Candidate kinases phosphorylating MARCKS at Ser46

(a) NetworKIN 3.0 was used to predict candidate kinases that phosphorylate MARCKS at Ser46. The top three include ERK1/2 and PKC mu.

(b) Summary of the mass spectrometric analysis of *in vitro* phosphorylation of the GST-MARCKS (1-176 aa) peptide by each kinase.

Supplementary Figure 9

Dynamism of HMGB1 in neurons with intracellular Aβ accumulation and after the rupture

(a) Co-staining of HMGB1 and Aβ revealed that cytoplasmic translocation of HMGB1 occurred in neurons with intracellular Aβ accumulation in the cerebral cortex of 5xFAD mice. Magnifications of fields #1-3 are shown in the lower panels. Field #2 might be an earlier phenotype than #3 based on the amount and distribution of intracellular Aβ. The graph shows quantification of cytoplasmic HMGB1-positive cells.

(b) HMGB1 did not bind to the ghost cells or extracellular Aβ aggregations.

(c) Three-dimensional images of the same visual field (arrowhead) also confirmed the findings.

Supplementary Figure 10

Chronological changes of MARCKS phosphorylation during ageing of 5xFAD mice

(a) Low magnification of sagittal sections of 5xFAD and wild type (B6/SJL) mice stained with an anti-pSer46-MARCKS antibody. At 3 and 6 months of age, the

plaque-like pattern of staining was detected. At 1 month of age (when not aggregated in the extracellular space), the plaque-like stains were not present, whereas fibril-like stains of pSer46-MARCKS were detected.

(b) Co-staining with Aβ revealed that the fibril-like stains at 1 month correspond to neural fibres among the cells. Moreover, the cytoplasm of neurons without intracellular Aβ was stained with pSer46-MARCKS, suggesting that a specific extracellular trigger rather than an intracellular trigger of Aβ was critical for MARCKS phosphorylation at Ser46.

Supplementary Figure 11

HMGB1 activates MARCKS phosphorylation at Ser46 through TLR4

(a) Time-dependent response of MARCKS phosphorylation at Ser46 after addition of 5 nM HMG to the culture medium of primary mouse cortical neurons prepared from the E15 embryonic cerebral cortex. Quantitative analyses are shown in graphs for MARCKS/GAPDH, pSer46-MARCKS/MARCKS and pSer46-MARCKS/GAPDH.

(b) HMGB1 dose-dependent response of MARCKS phosphorylation at Ser46 in mouse primary cortical neurons (E15). The responses were evaluated 180 minutes after the addition of HMGB1 to the culture medium. Quantitative analyses are shown in the graphs.

(c) The response of MARCKS phosphorylation at Ser46 was specific to HMGB1. Addition of BDNF did not induce MARCKS phosphorylation.

(d) Effects of various Aβ species on MARCKS phosphorylation at Ser46. Aβ monomers, oligomers and aggregates were added at 25 nM to the medium of the primary mouse cortical neurons. Slight activation was observed only with the Aβ oligomer.

(e) A mixture of HMGB1 and Aβ triggered MARCKS phosphorylation at Ser46.

(f) Similar MARCKS phosphorylation at Ser46 was induced by a TLR4 agonist (LPS-EB).

(g) HMGB1-induced MARCKS phosphorylation at Ser46 was blocked by a TLR4 antagonist (LPS-RS).

(h) HMGB1-induced MARCKS phosphorylation at Ser46 was blocked by knockdown of TLR4 with shRNA.

Supplementary Figure 12

Selection of the best anti-HMGB1 antibody

(a, b) Seven clones of the anti-HMGB1 antibody were compared for their affinity to HMGB1. ELISA **(a)** and surface plasmon resonance (SPR) analysis **(b)** were used to evaluate the affinity of antibodies to HMGB1. We selected the clone 2C8C because it showed relatively high affinity in both the ELISA and SPR analyses.

Supplementary Figure 13

Transfer of the antibody from subcutaneous injection to the brain tissue

(a) To evaluate the efficiency of a subcutaneous injection (SC) in delivering the antibody to the brain, we injected biotin-labelled IgG using a similar method to that of the anti-HMGB1 antibody and examined the concentrations in blood plasma and in brain tissues. Some of the injected IgG (0.008%) was present in the brain tissue that had been prepared after systemic perfusion with PBS.

(b) Immunohistochemistry with avidin-HRP and DAB colour development was performed to detect biotin-labelled mouse IgG in the brain tissues, but no definite signal was detected.

Supplementary Figure 14

***In vivo* effects of the subcutaneous injection of the anti-HMGB1 antibody on MARCKS phosphorylation at Ser46 and on Aβ plaque formation**

(a) In both groups of 5xFAD mice that had received subcutaneous injection of the anti-HMGB1 monoclonal antibody during 1-6 or 3-6 months, the signal intensity of the plaque-like stain pattern of pSer46-MARCKS was substantially decreased compared to the group that had received a subcutaneous injection of non-specific IgG (upper panels). The number of Aβ plaques was mildly decreased by subcutaneous injection of the anti-HMGB1 monoclonal antibody (lower panels). The inlays show a few plaque-like stains.

(b) Aβ signal intensity in immunohistochemistry by DAB colour development also confirmed that Aβ plaques were mildly decreased by subcutaneous injection of the anti-HMGB1 monoclonal antibody. The lower graphs show the

effect of anti-HMGB1 monoclonal antibody on the number and area of Aβ plaques.

(c) In contrast to immunohistochemistry, Western blot analysis revealed a reduction in the total amount of Aβ by the anti-HMGB1 monoclonal antibody (upper panels), especially oligomers and protofibrils (lower graph).

(d) Western blot analysis revealed that the anti-HMGB1 monoclonal antibody decreased pSer46-MARCKS and γH2AX, a DNA damage marker.

(e) Western blot and qPCR analyses showed that human APP gene expression was not changed by anti-HMGB1 monoclonal antibody.

Supplementary Figure 15

Experimental conditions for *in vitro* Aβ aggregation

(a) Experimental conditions for *in vitro* Aβ aggregation are shown in the scheme. Sampling was performed before and after incubation.

(b) Western blot analysis of *in vitro* Aβ aggregation. Two types of sample buffer were tested, and six conditions were tested. We chose one condition (incubation at 37°C for 48 hours) because additional incubation did not affect the aggregation, and we used mercaptoethanol (-) sample buffer for further analyses.

(c) Electron microscopic analysis of *in vitro* Aβ aggregation also revealed that the condition was suitable for further analyses.

Supplementary Figure 16

***In vitro* effects of HMGB1 and anti-HMGB1 antibody on Aβ polymerization, Aβ oligomerization, Aβ-HMGB1 heteromer formation and HMGB1 oligomerization**

(a) Aβ was incubated for 48 hours with or without HMGB1 and with or without the HMGB1 antibody. Normal mouse IgG was used as a control. HMGB1 notably decreased fibrils/aggregates of Aβ and increased the other types of Aβ species, such as monomers, oligomers and ADDLs/protofibrils. Further addition of the anti-HMGB1 antibody suppressed the HMGB1-induced decrease of Aβ fibrils/aggregates and inhibited the HMGB1-induced increase of Aβ oligomers and ADDLs/protofibrils.

(b) Quantitative analysis of ratios among four types of Aβ species in the total WB signals of Aβ (IgG signals were excluded). Mean values of three experiments were used.
(c) The effects of HMGB1 and the anti-HMGB1 antibody on Aβ oligomerization were further investigated. HMGB1 clearly increased the Aβ monomer, oligomer and ADDLs/protofibrils in a dose-dependent manner. The anti-HMGB1 antibody but not the control IgG blocked the effect of HMGB1 on Aβ oligomerization.
(d) Using the same sample, *in vitro* polymerization of HMGB1 was detected. Aβ inhibited the HMGB1 polymerization and produced other types of Aβ-HMGB1 heteromers as determined by their molecular weight.
(e) In the absence of Aβ, HMGB1 aggregation was promoted in a dose-dependent manner. The anti-HMGB1 antibody inhibited HMGB1 polymerization and increased the ratio of HMGB1 monomers and dimers. This effect was not observed with the control IgG.
(f) Electron microscopic observation of *in vitro* aggregation samples. HMGB1 blocked fibril formation of Aβ (black arrows) and increased amorphous structures that might correspond to Aβ oligomers or ADDLs/protofibrils. Addition of the anti-HMGB1 antibody recovered fibril formation (black arrows). Immunoelectron microscopy revealed that HMGB1 was located in the periphery (white arrows), presumably blocking extension of the Aβ fibrils.

Supplementary Figure 17

***In vivo* effect of the anti-HMGB1 monoclonal antibody on microglia in 5xFAD mice**

(a) Low magnification of the cerebral cortex (RSD) and high magnification of a representative Aβ plaque of 5xFAD mice at 6 months of age are shown. After treatment with the anti-HMGB1 antibody (both the 1-6 months and 3-6 months treatment groups), a larger number of microglia formed Aβ plaques, and the microglia incorporated Aβ into the cytoplasm.
(b) Quantitative analysis of the number of microglia in a visual field (N=10, cerebral cortex) and the number of microglia forming plaques (N=30, cerebral cortex). Both values were increased in the mice after treatment with the anti-HMGB1 antibody.

(c) Phagocytosis of fluorescent Aβ by rat microglia in primary cultures. TAMRA-Aβ, human HMGB1 and anti-HMGB1 monoclonal antibodies were mixed, pre-incubated and added to the primary microglial culture (upper panel). Addition of the anti-HMGB1 monoclonal antibody enhanced phagocytosis of TAMRA-Aβ ((middle photos)), and quantitative analysis confirmed the findings (lower graph).

Supplementary Figure 18

Aβ species at high concentrations trigger the release of HMGB1 from neurons

Monomers, oligomers and fibrils of Aβ were added to the primary mouse cortical neuron cultures prepared from E15. After 3 hours, HMGB1 concentrations in the culture medium were examined by ELISA.

Supplementary Figure 19

Hypothetical mechanisms of the therapeutic effects of the anti-HMGB1 monoclonal antibody

(a) Pathological status of AD focusing on Aβ and HMGB1. **(b)** Ameliorated pathological status of AD after injection of the anti-HMGB1 monoclonal antibody.

Supplementary Video 1

3D movie of co-staining of pSer46-MARCKS staining and Aβ in 6-month-old 5xFAD mice cortex.

This movie corresponds to the image in figure 2B.

Supplementary Video 2

3D movie of co-staining of pSer46-MARCKS staining and Aβ in human AD brain.

This movie corresponds to the image in figure 2C.

Supplementary Video 3

Relationship between pSer46-MARCKS staining and Aβ aggregation in the transparent brain of human APP knock-in mice.

(a) This movie corresponds to the image of 8-month-old human APP knock-in mice brain in Supplementary figure 5B.

(b) This movie corresponds to the image of 17-month-old human APP knock-in mice brain in Supplementary figure 5B.

(c) This movie corresponds to the image of 20-month-old human APP knock-in mice brain in Supplementary figure 5B.

Supplementary Video 4

3D movie of co-staining of pSer46-MARCKS staining and Tau in human AD brain.

This movie corresponds to the image in figure 2D.

Supplementary Figure 1

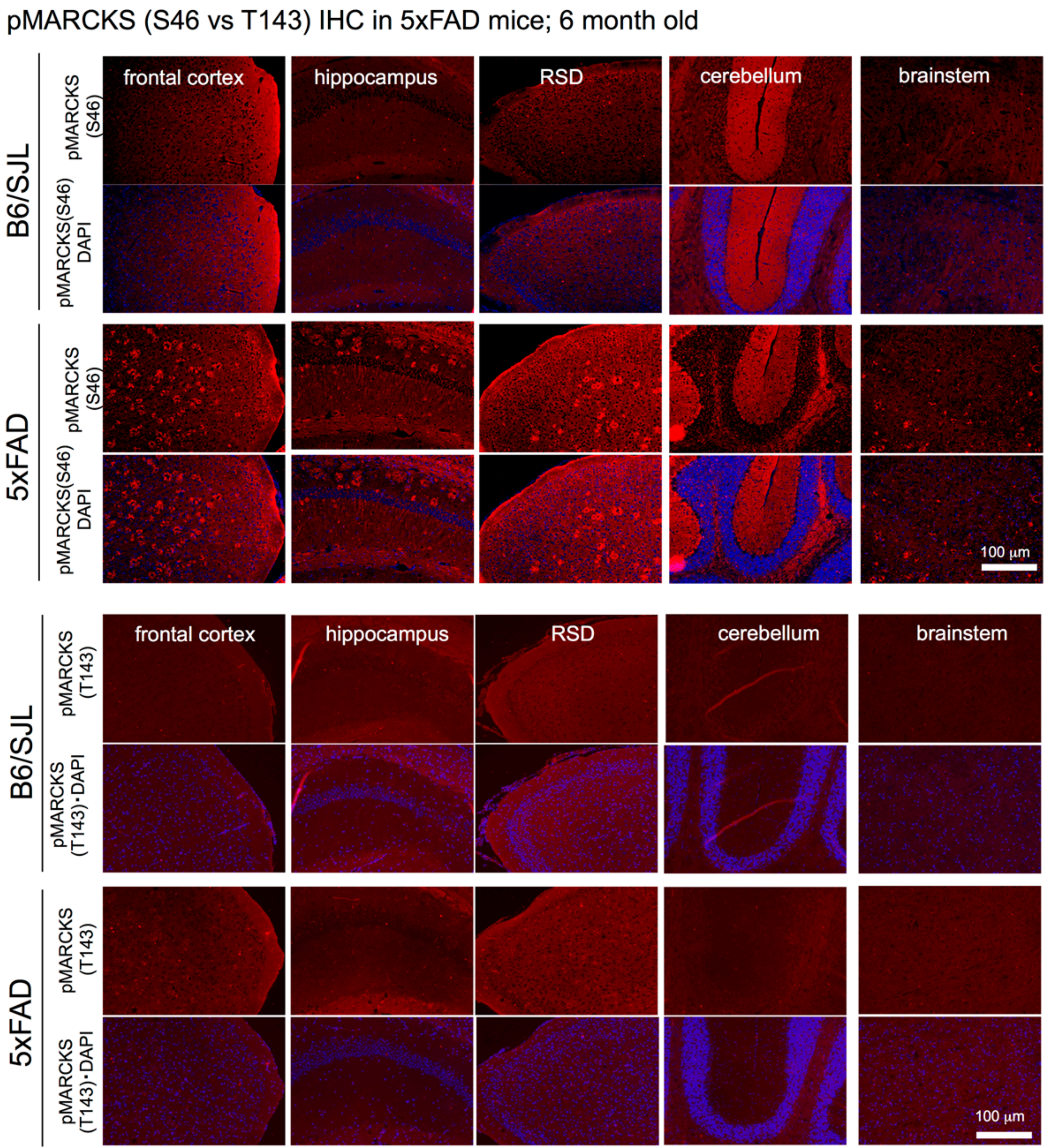

Supplementary Figure 2

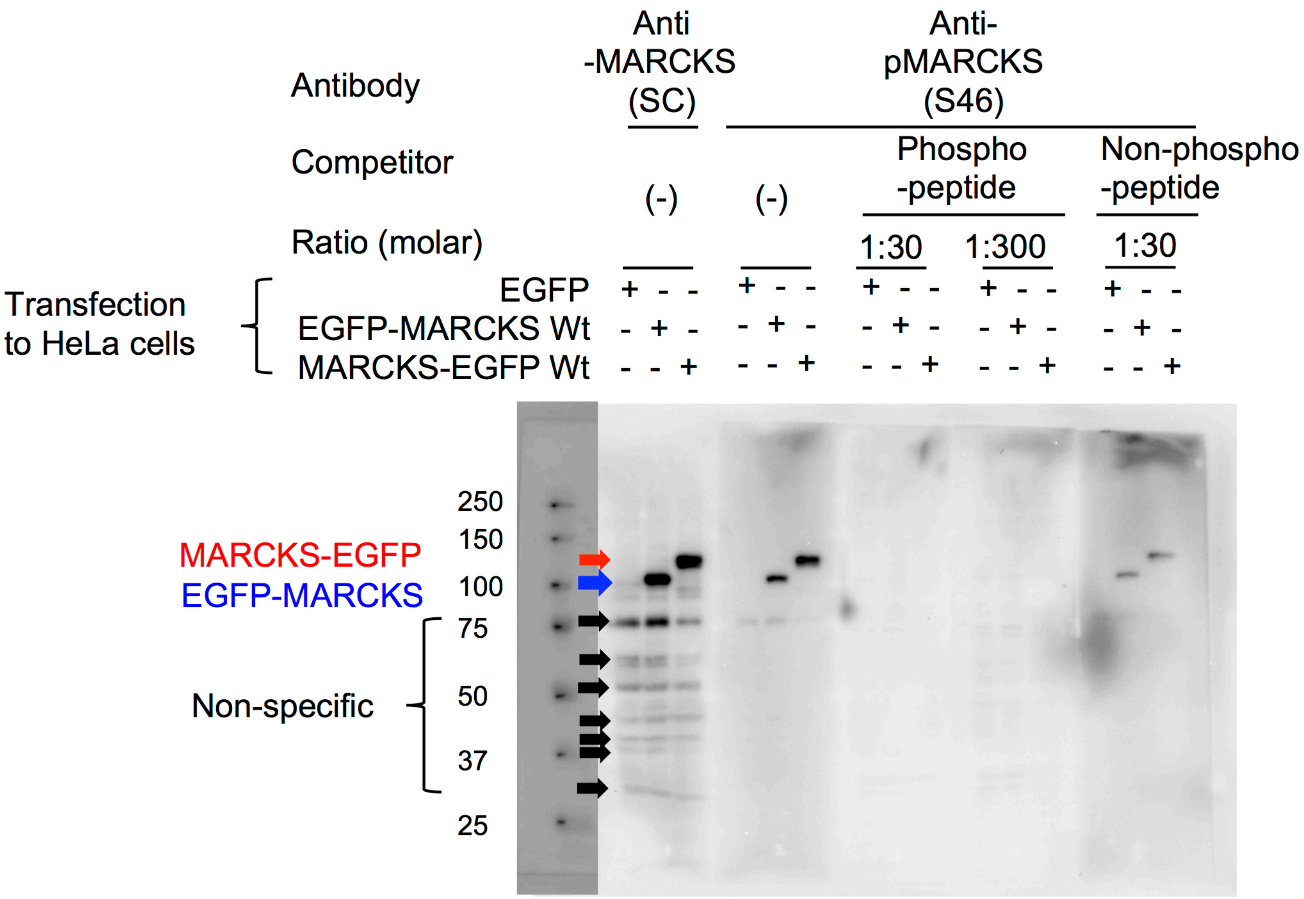

SC:Anti-full-length MARCKS (SantaCrus)
1/1000

2nd Ab GE anti-Mouse
1/3000

Anti-pMARCKS (S46)
(Original Lot. RB5871)
1/100,000

Antigen sequence
1. Phospho-peptide (ENGHVKVNGDA(pS)PA)
2. Non-phospho-peptide (ENGHVKVNGDASPA)

2nd Ab GE anti-Mouse
1/3000

Supplementary Figure 3

5xFAD Mouse
Aβ and pS46-MARCKS

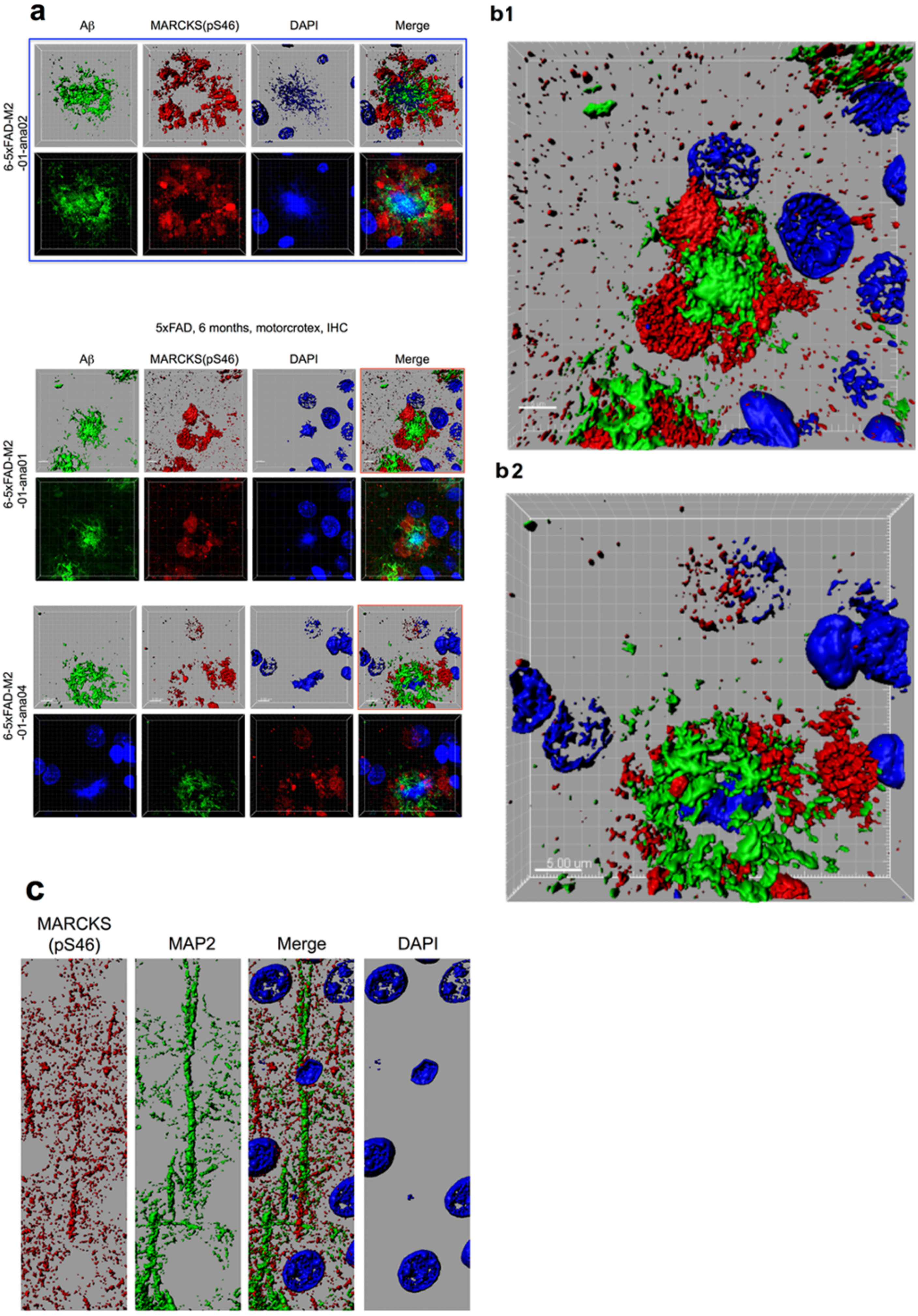

Supplementary Figure 4

Human AD
Aβ and pS46-MARCKS

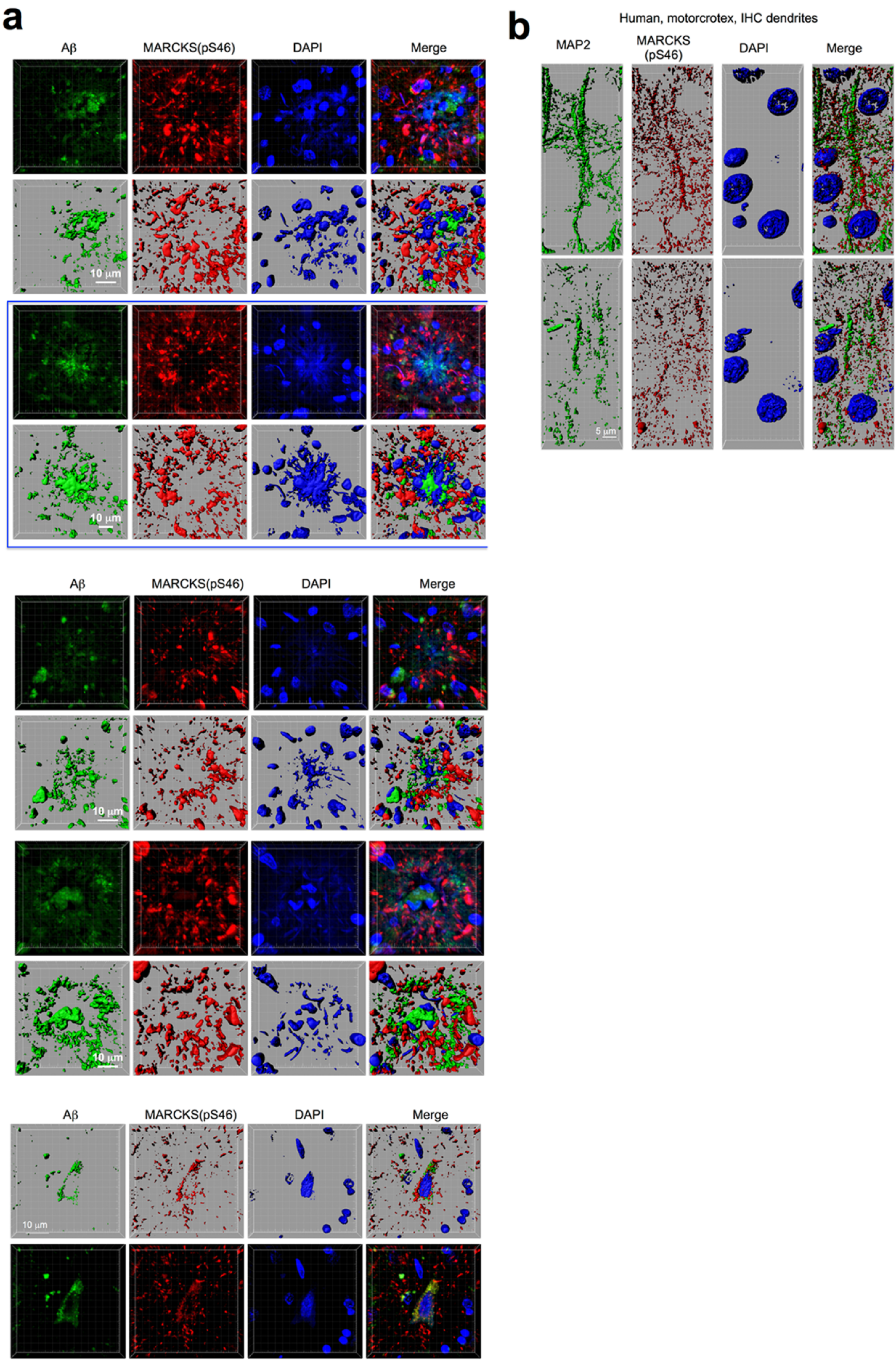

Supplementary Figure 5

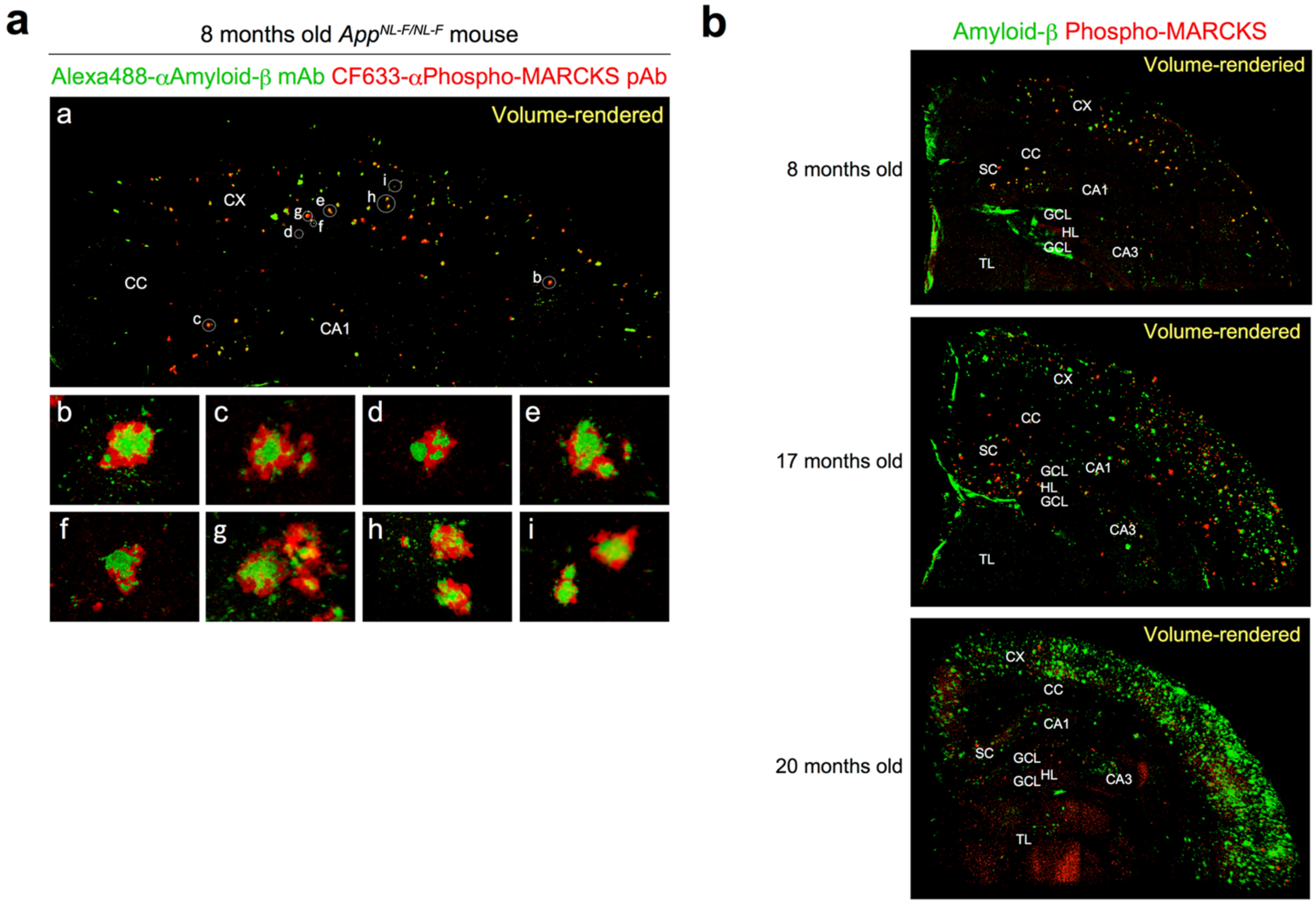

CC: corpus callosum, CX: cortex, GCL: granule cell layer, HL: hilus, SC:subiculum, TL: thalamus

Supplementary Figure 6

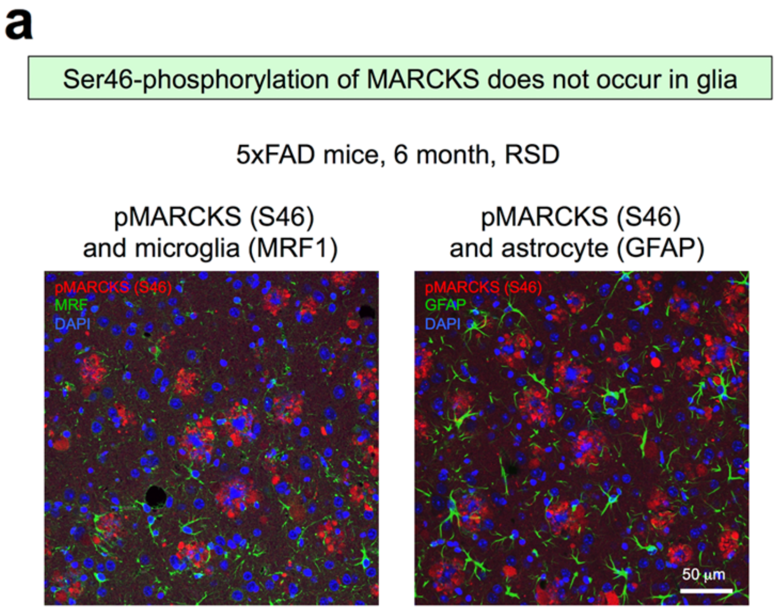

MRF: microglial response factor 1
GFAP: glial fibrilary acidic protein

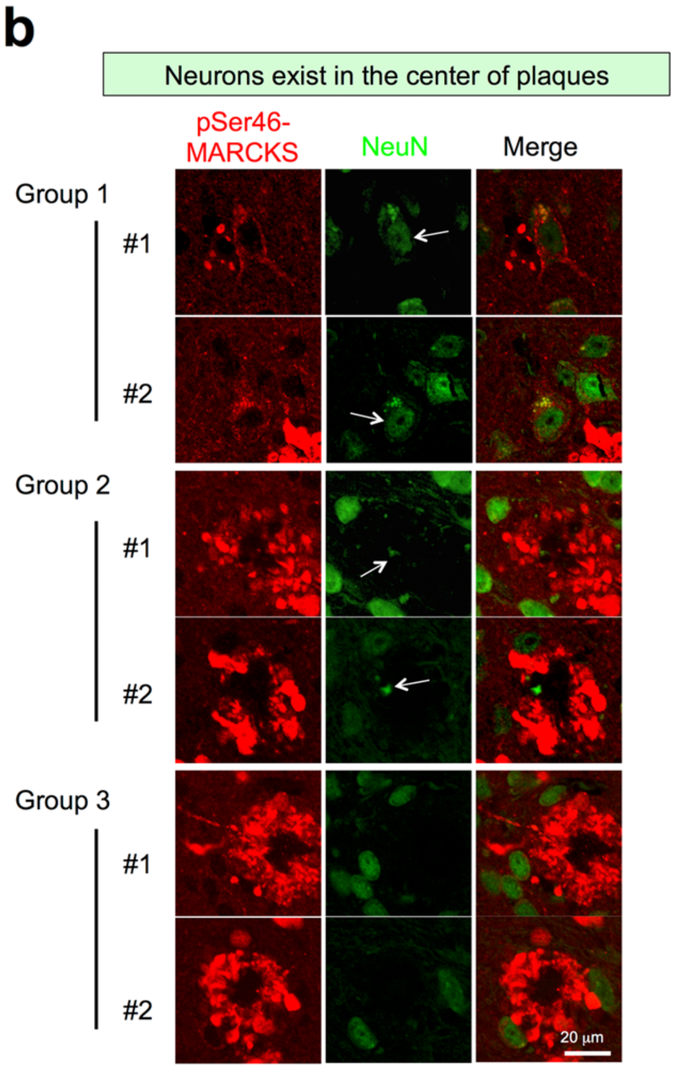

Supplementary Figure 7

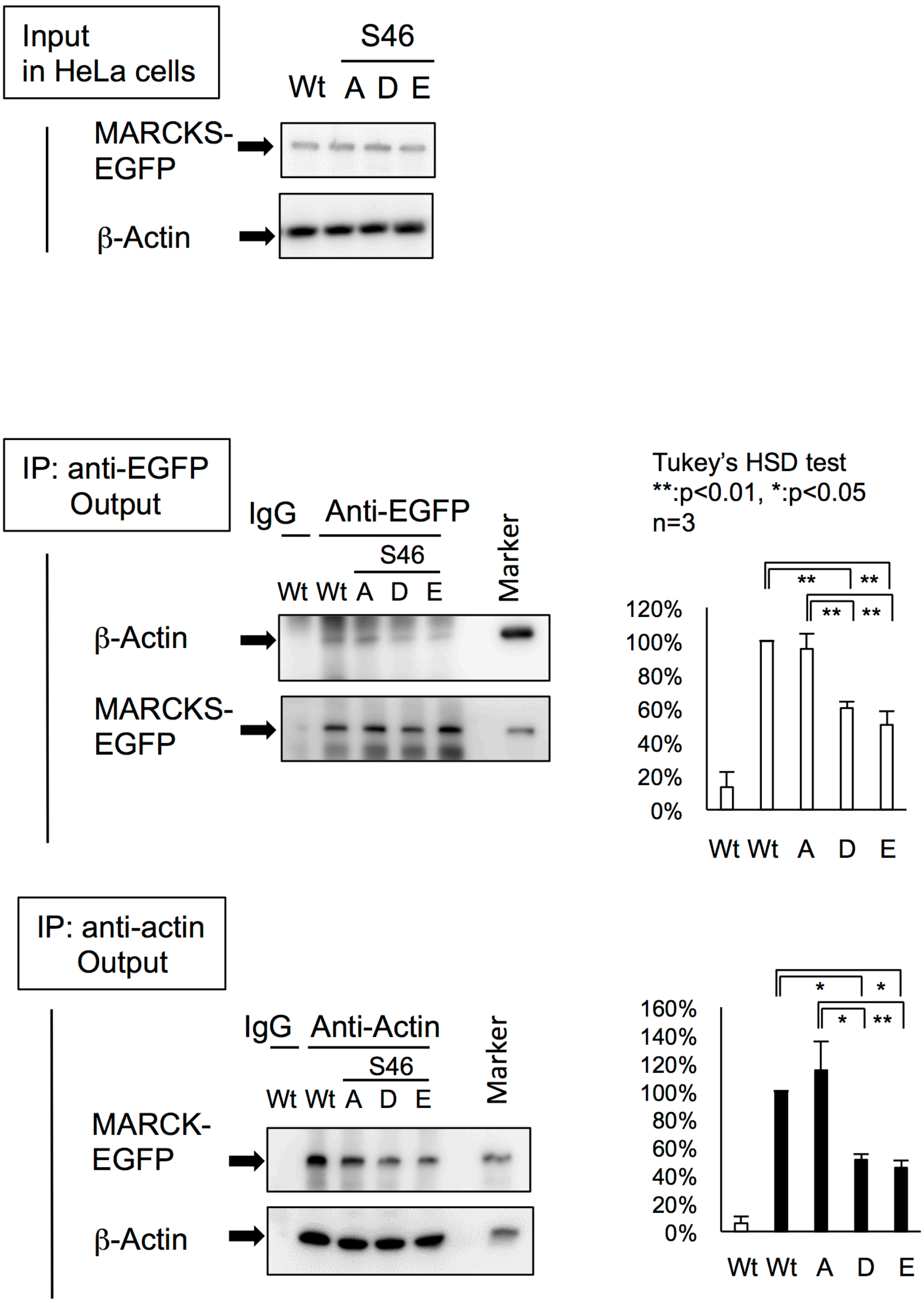

Supplementary Figure 8

a

Networkin 3.0
MARCKS S46
string_identifier : ENSP00000357624

id	Name	networkin score	netphorest group	netphorest score	string score
MAPK1	ERK2	3.06098	MAPK3_MAPK1_MAPK7_NLK_group	0.105618	0.818
PKD1	PKC-mu	2.27591	PKD_group	0.039228	0.564
MAPK3	ERK1	0.983546	MAPK3_MAPK1_MAPK7_NLK_group	0.105618	0.407673
MAPK12	p38 γ	0.351341	p38_group	0.14822	0.234388
MAPK13	p38 δ	0.348053	p38_group	0.14822	0.209543
MAPK11	p38 β	0.347193	p38_group	0.14822	0.203048
CDK1		0.332751	CDK2_CDK3_CDK1_CDK5_group	0.074269	0.381842
MAPK10	JNK3	0.307479	JNK_group	0.121194	0.183229
GSK3beta		0.295009	GSK3_group	0.044283	0.331011
NLK		0.283417	MAPK3_MAPK1_MAPK7_NLK_group	0.105618	0.162954
MRCKa		0.282431	DMPK_group	0.048257	0.372
MAPK8	JNK1	0.259509	JNK_group	0.121194	0.288365
PKD3		0.258335	PKD_group	0.039228	0.36
GSK3alpha		0.232009	GSK3_group	0.044283	0.344139
CDK3		0.224046	CDK2_CDK3_CDK1_CDK5_group	0.074269	0.144787
MOK		0.186607	RCK_group	0.042254	0.296768
DMPK2		0.165869	DMPK_group	0.048257	0.124167
DMPK1		0.165869	DMPK_group	0.048257	0.175951
MRCKb		0.165869	DMPK_group	0.048257	0.063374
CLK1		0.164885	CLK_group	0.043101	0.198142
CLK2		0.164756	CLK_group	0.043101	0.15297
CLK3		0.164756	CLK_group	0.043101	0.110813
CLK4		0.164756	CLK_group	0.043101	0.108364
ICK		0.164573	RCK_group	0.042254	0.141656
MAK		0.164573	RCK_group	0.042254	0.12918
HIPK1		0.164525	HIPK1_HIPK2_group	0.042031	0.131697
PKD2		0.164029	PKD_group	0.039228	0.156382
CDK2		0.118088	CDK2_CDK3_CDK1_CDK5_group	0.074269	0.345993
CDK5		0.026452	CDK2_CDK3_CDK1_CDK5_group	0.074269	0.326783
MAPK9	JNK2	0.000282	JNK_group	0.121194	0.312636
DNAPK		0.000153	DNAPK	0.050934	0.288717
MAPK7	ERK5	0.000128	MAPK3_MAPK1_MAPK7_NLK_group	0.105618	0.345122
HIPK2		0.000126	HIPK1_HIPK2_group	0.042031	0.161236
MAPK14	p38 α	0.000103	p38_group	0.14822	0.313981

b

				Peptide intensity					
Peptide detected by Mass Spec	Precursor MZ	Precursor Charge	Retention Time	ERK1	ERK2	JNK1	JNK2	JNK3	No kinase
Phosphorylated peptides at S46									
VN[Dea]GDAS[Pho]PAAAESGAK	713.298	2	22.7159	185.43	344.34	292.487	457.653	158.935	101.177
VN[Dea]GDAS[Pho]PAAAESGAK[Frm]	727.296	2	23.2915	211.915	242.506	158.93	52.97	185.415	174.771
AS[Pho]PAAAESGAK	520.226	2	16.4778	105.96	79.47	79.47	52.97	105.94	52.98
VN[dAm]GDAS[Pho]PAAAESGAK	704.293	2	24.1727	132.45	1117.64	2594.11	1438.6	1900.5	79.47
VN[Dea]GDAS[Pho]PAAAESGAK[CEt]	749.309	2	24.4741	182.116	105.955	147.2	105.94	52.98	156.573
VN[Dea]GD[NaX]AS[Pho]PAAAESGAK	724.289	2	22.0554	211.92	79.455	175.673	105.94	181.999	158.91
VN[Dea]GD[KXX]AS[Pho]PAAAESGAK	732.276	2	22.0775	211.92	185.395	175.673	132.43	52.98	105.94
Non-phosphorylated peptides at Ser46									
VN[Dea]GDASPAAAESGAK	673.315	2	22.4476	79.44	185.395	271.496	264.89	278.1	638.339
VN[Dea]GDAS[Pho]PAAAESGAK / VN[Dea]GDASPAAAESGAK			P / non P	2.33	1.86	1.08	1.73	0.57	0.16
Ratio compared to no kinase (folds)				14.56	11.63	6.75	10.81	3.56	

Supplementary Figure 9

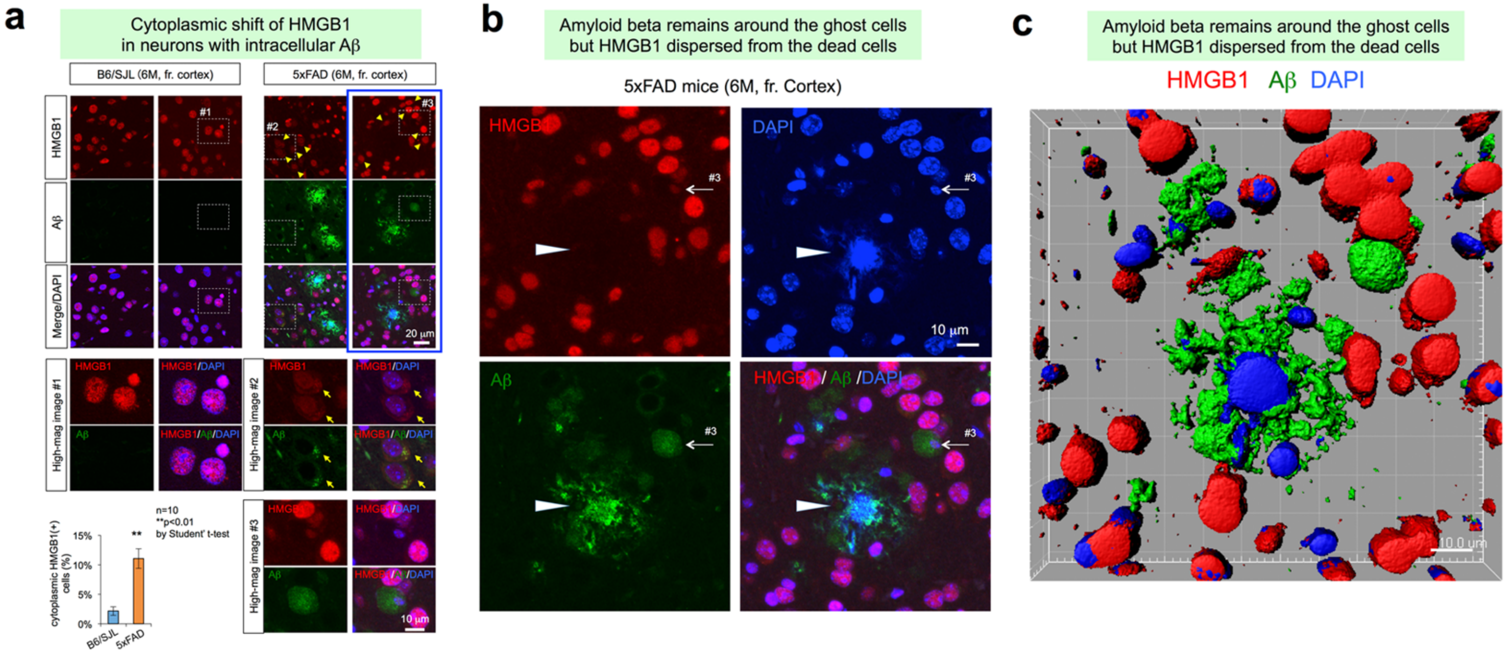

Supplementary Figure 10

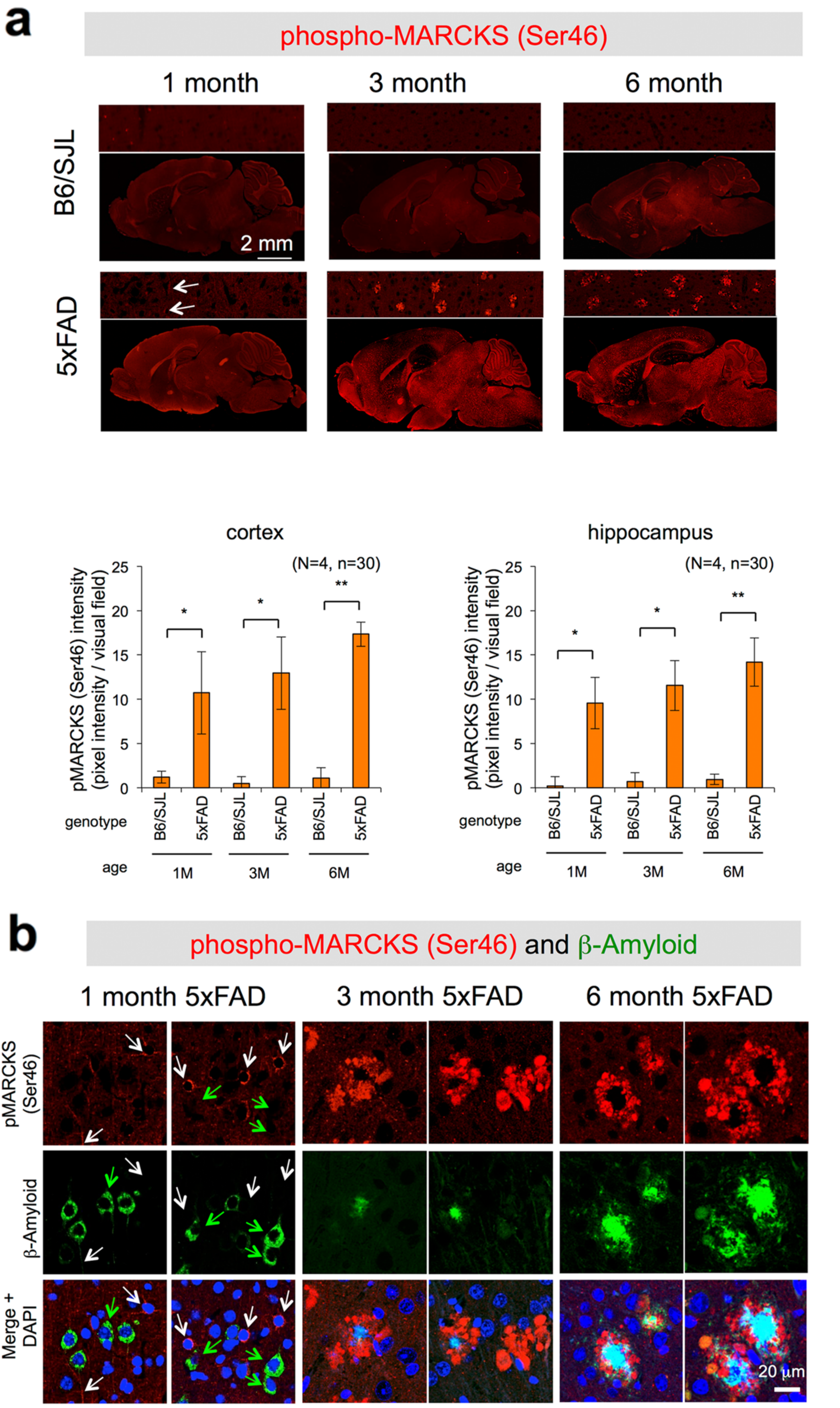

Supplementary Figure 11

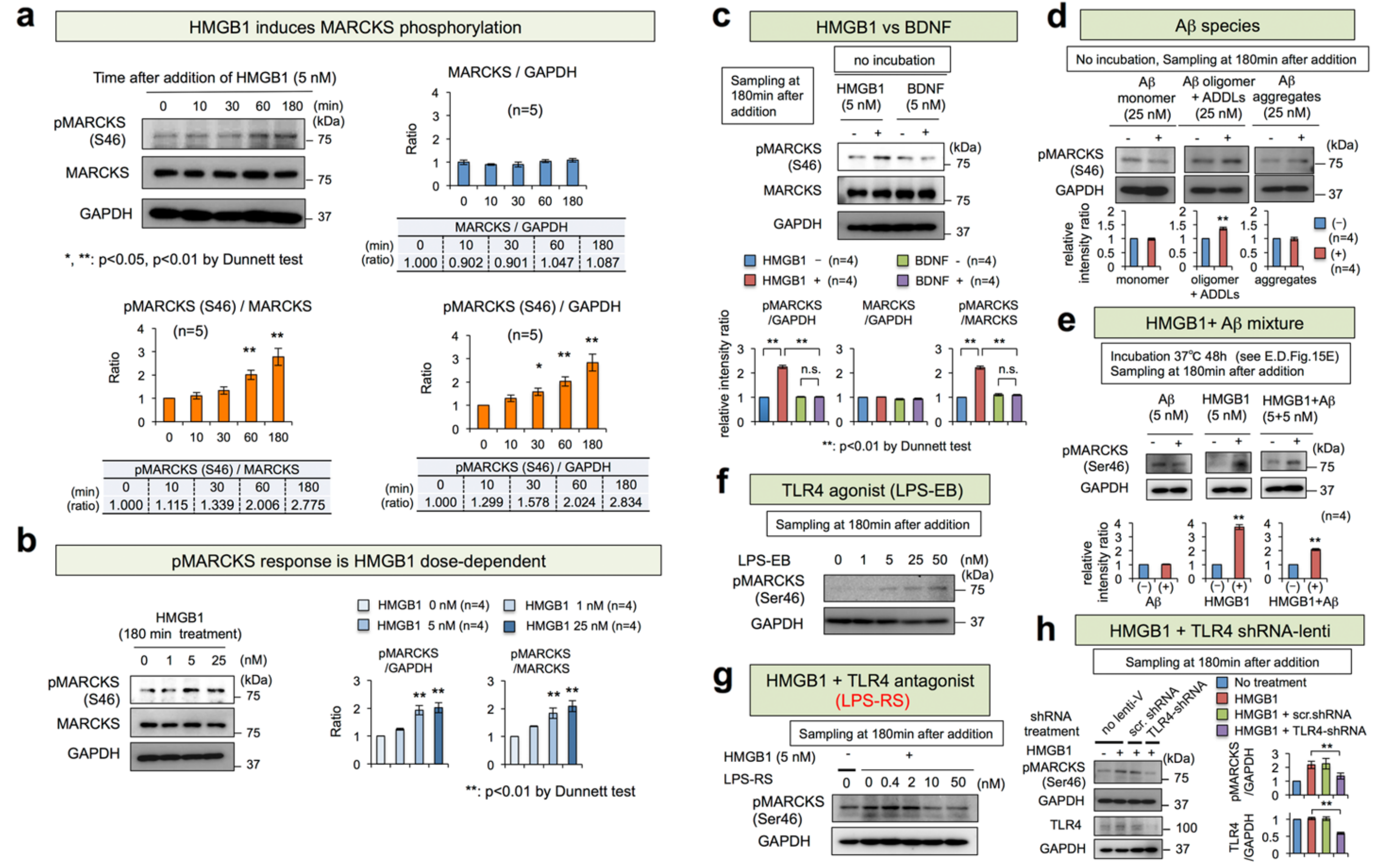

	MARCKS / GAPDH				
(min)	0	10	30	60	180
(ratio)	1.000	0.902	0.901	1.047	1.087

	pMARCKS (S46) / MARCKS				
(min)	0	10	30	60	180
(ratio)	1.000	1.115	1.339	2.006	2.775

	pMARCKS (S46) / GAPDH				
(min)	0	10	30	60	180
(ratio)	1.000	1.299	1.578	2.024	2.834

Supplementary Figure 12

a

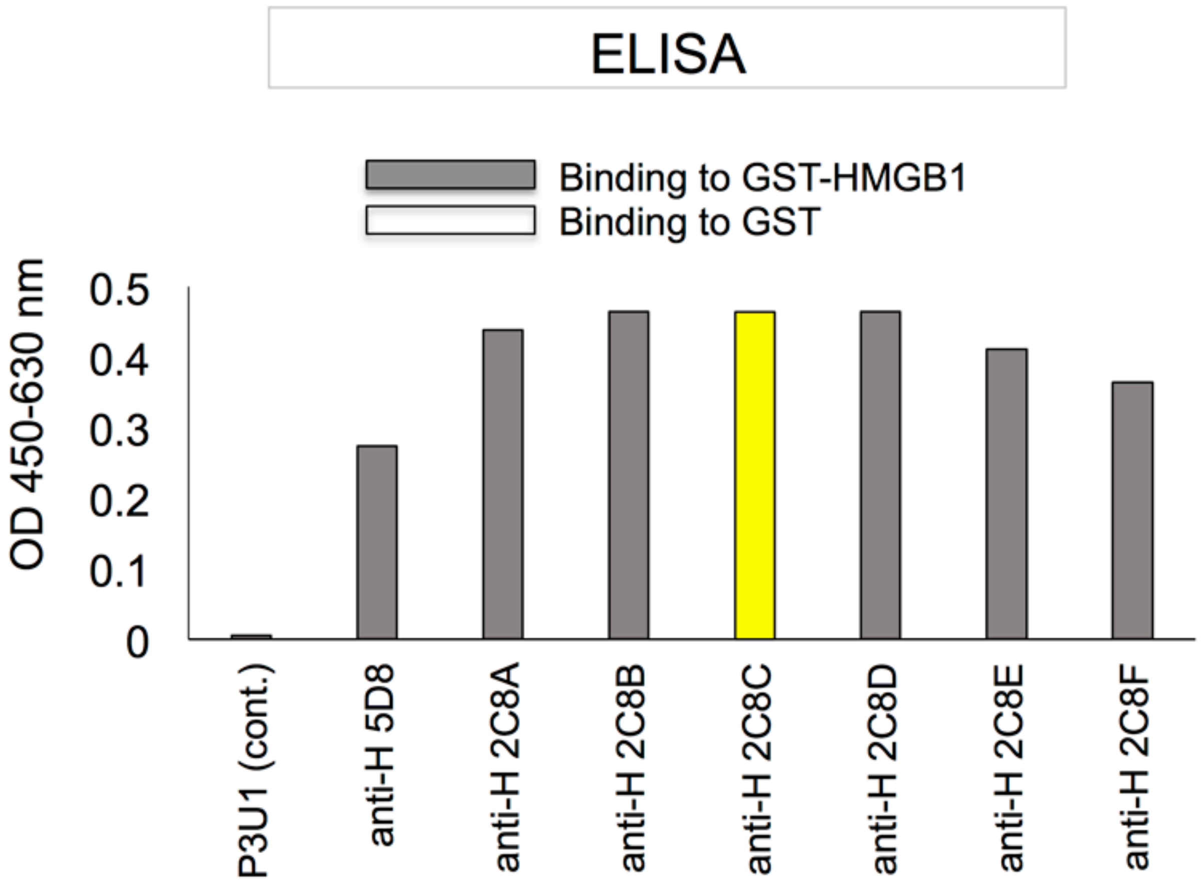

b

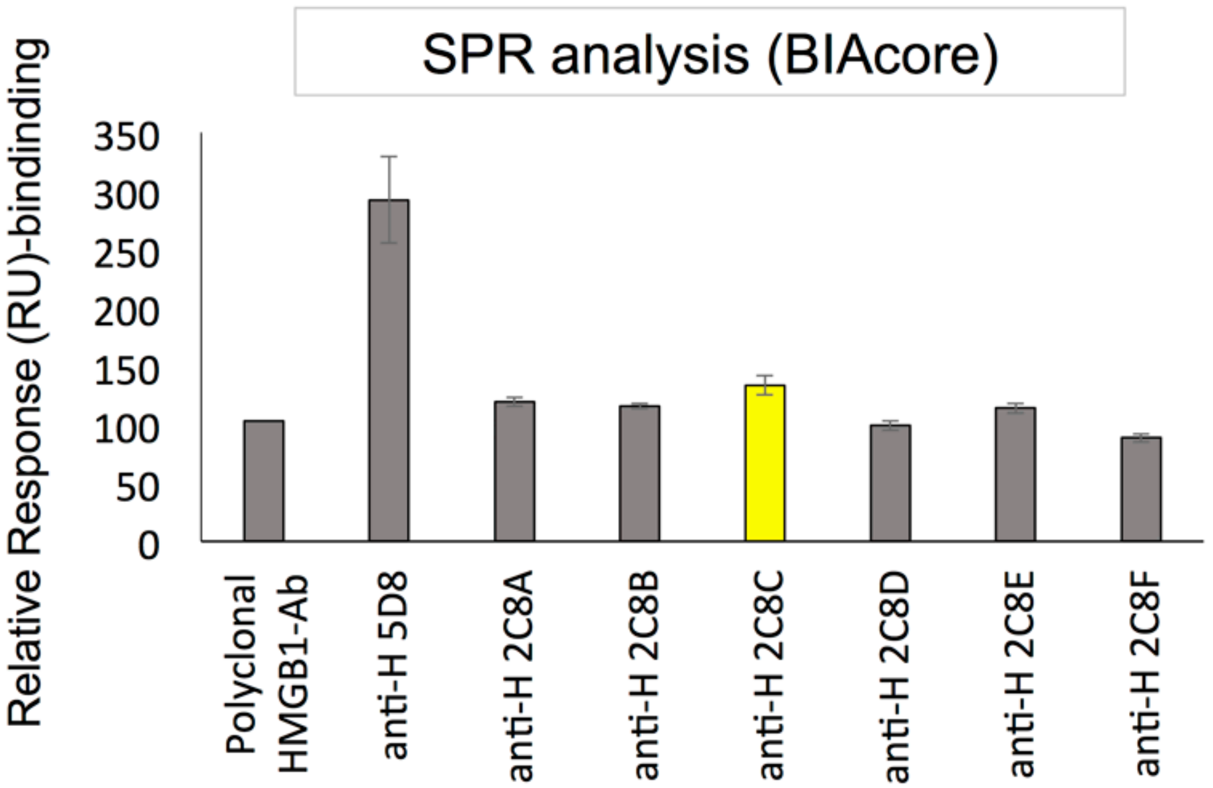

Supplementary Figure 13

a

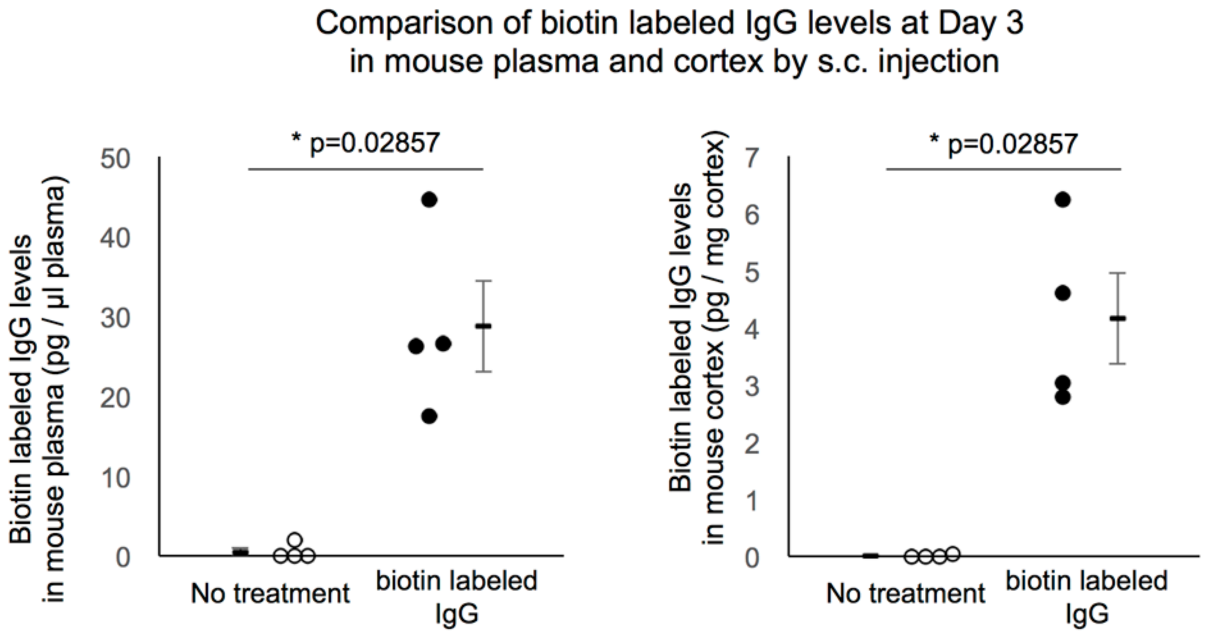

○: Lower detection limit.
*: p-values by Wilcoxon rank sum test and Fisher's exact test. (Both p-values were equivalent.)

b

C57BL/6j male 2 months old
Biotin-labeled mouse IgG 1mg/kg
SC injection

day1 or day3 after injection
n=3

PFA-fixed, paraffin-embedded 5um

Biotin-detect:
Avidin-HRP+ DAB color development

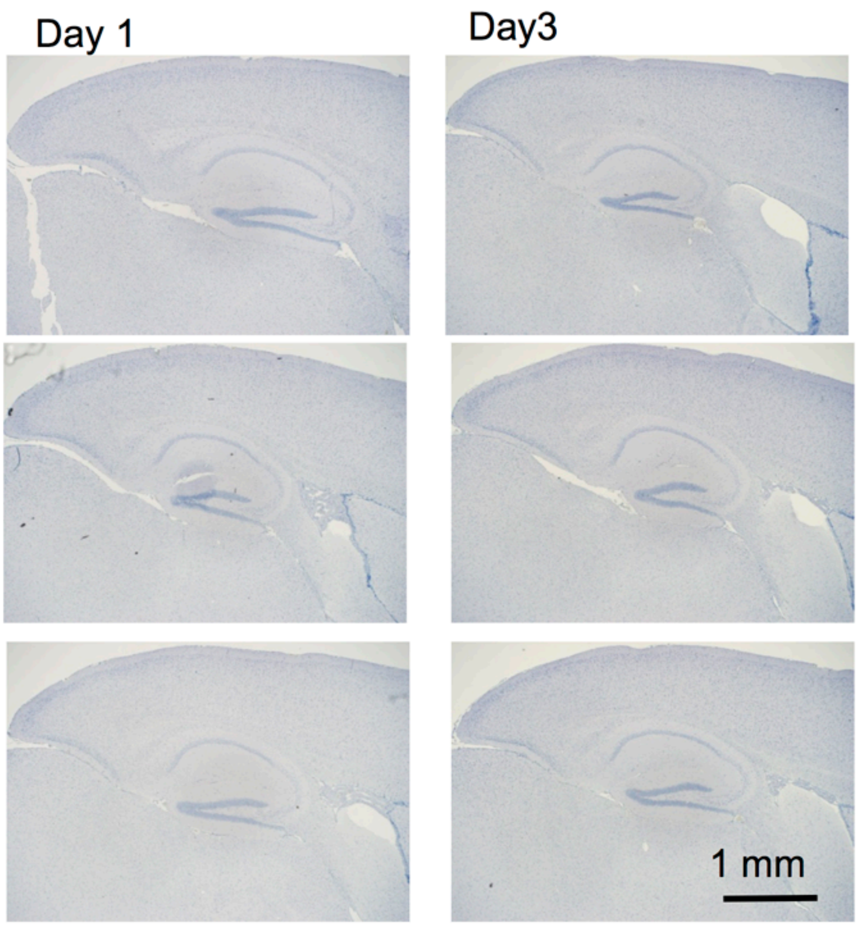

Supplementary Figure 14

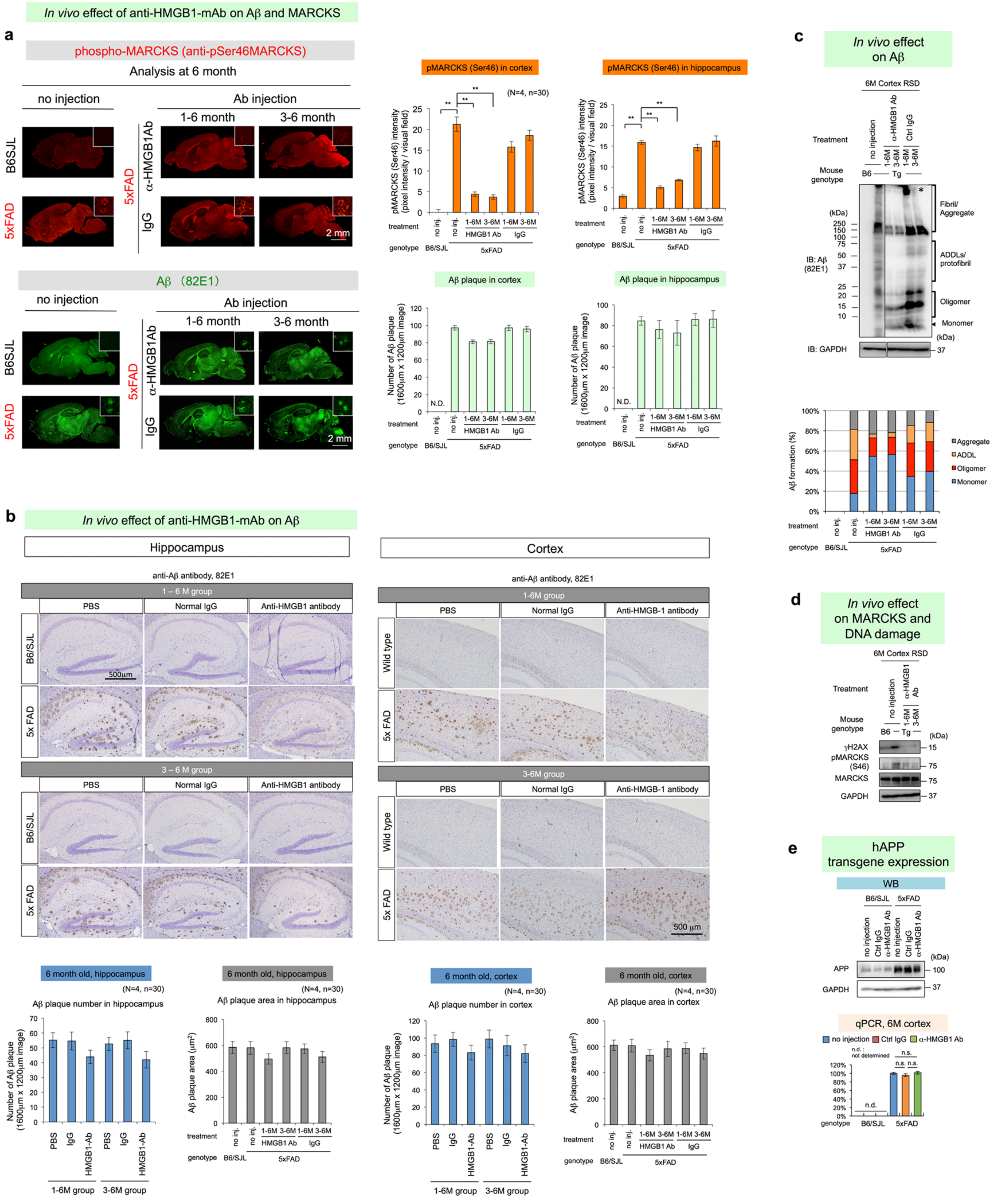

Supplementary Figure 15

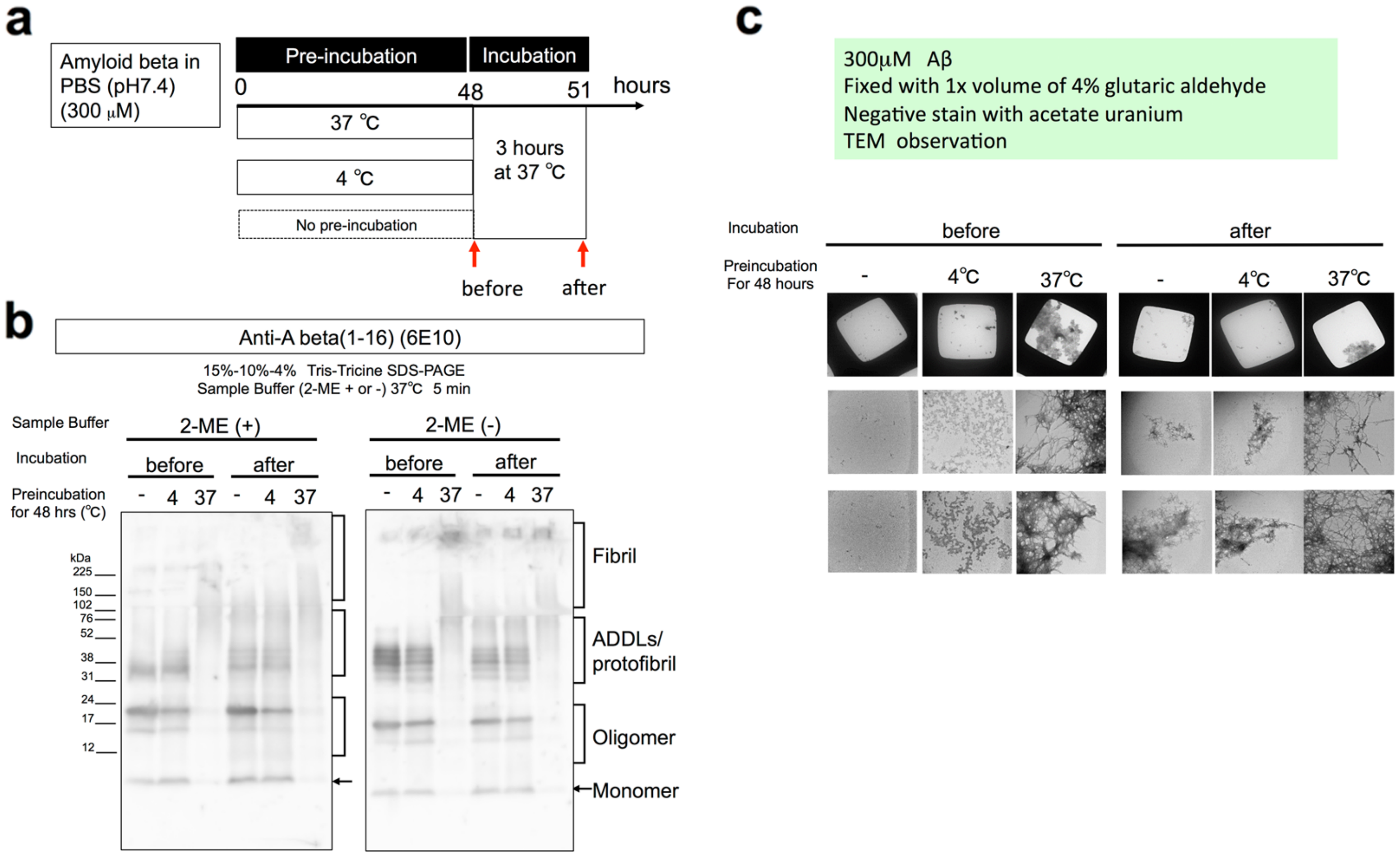

Supplementary Figure 16

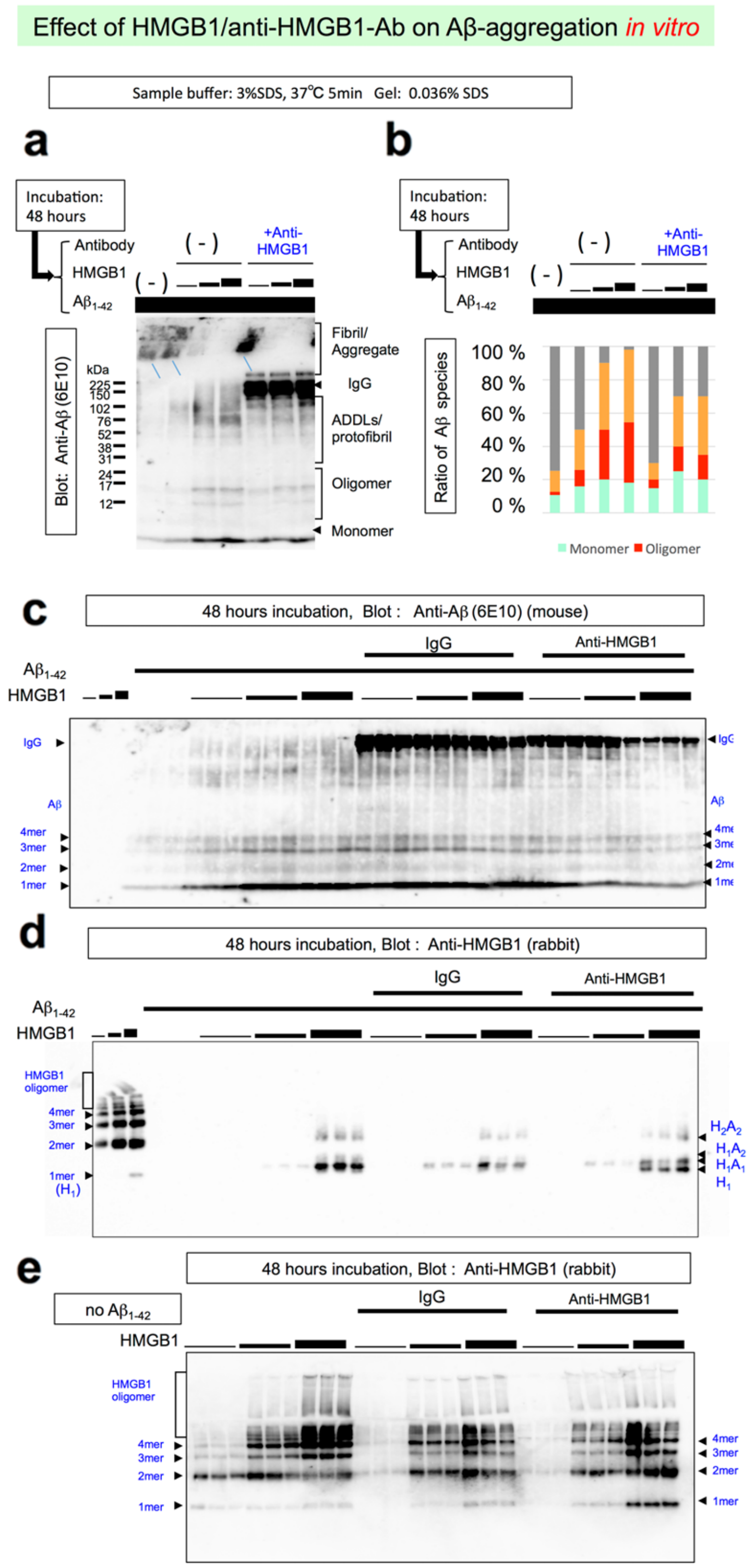

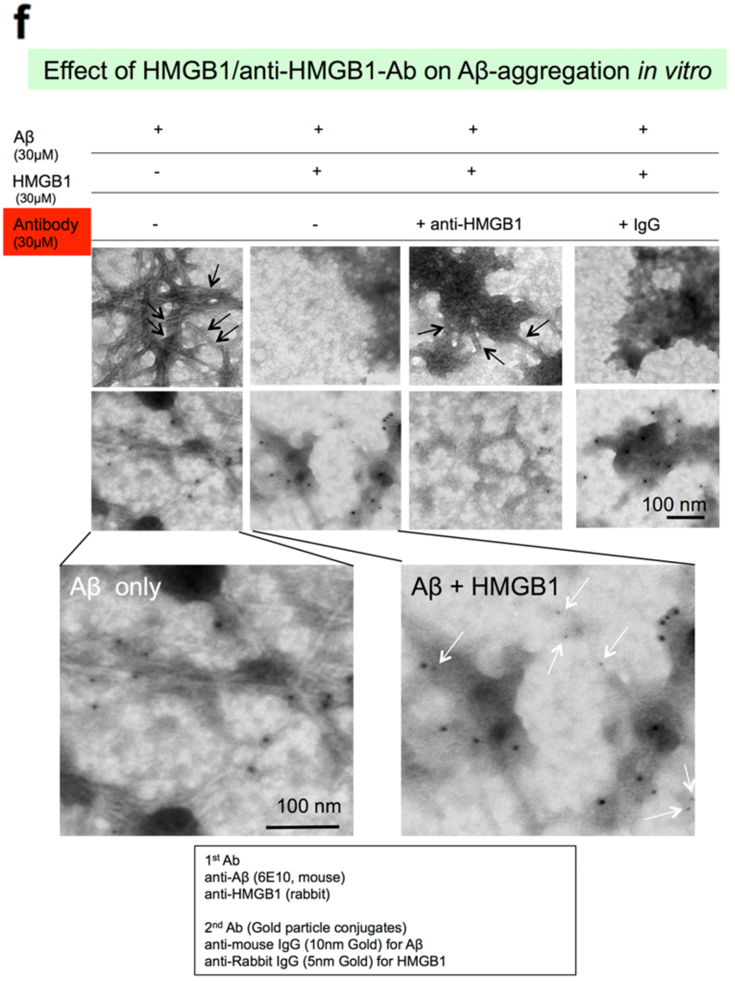

Supplementary Figure 17

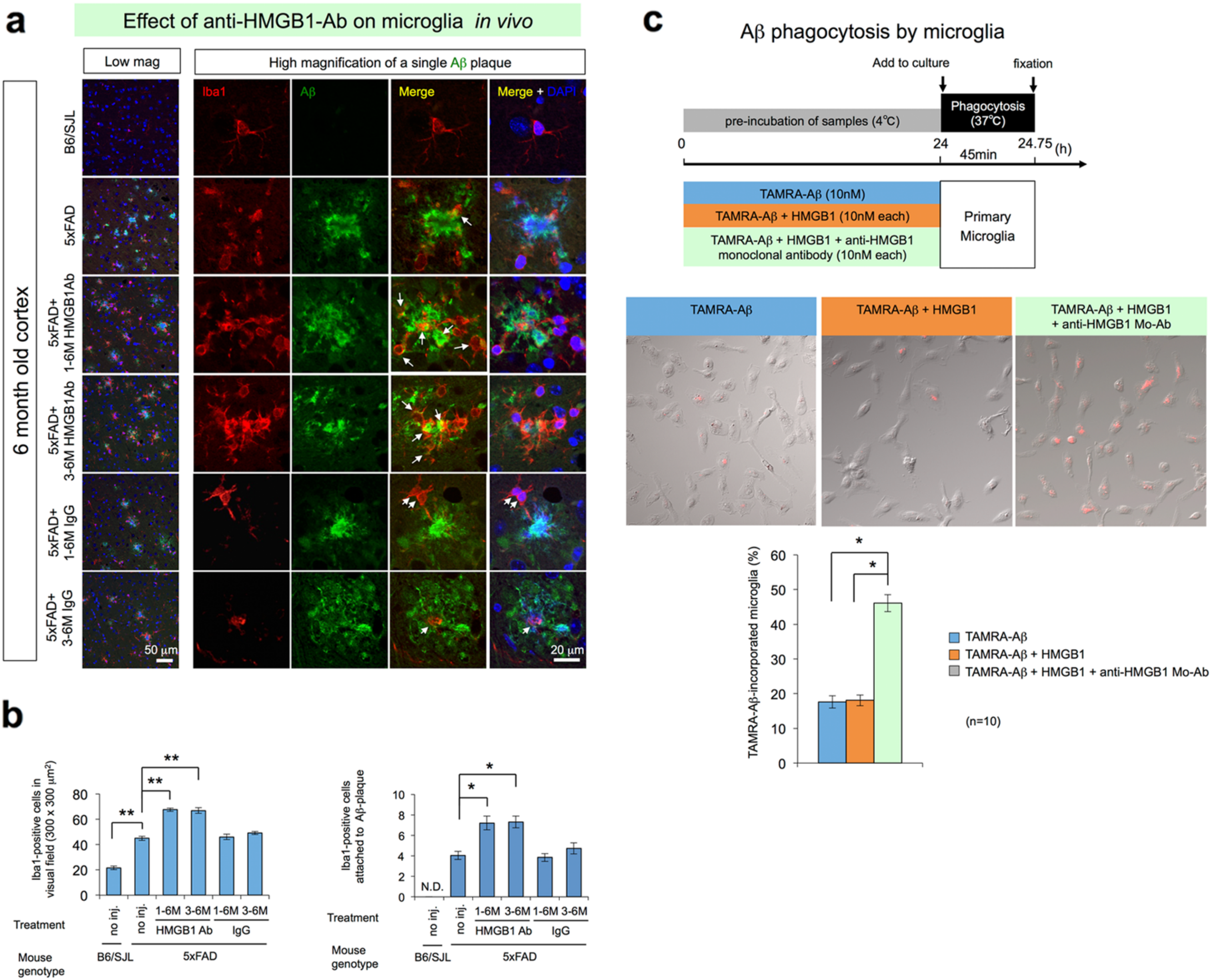

Supplementary Figure 18

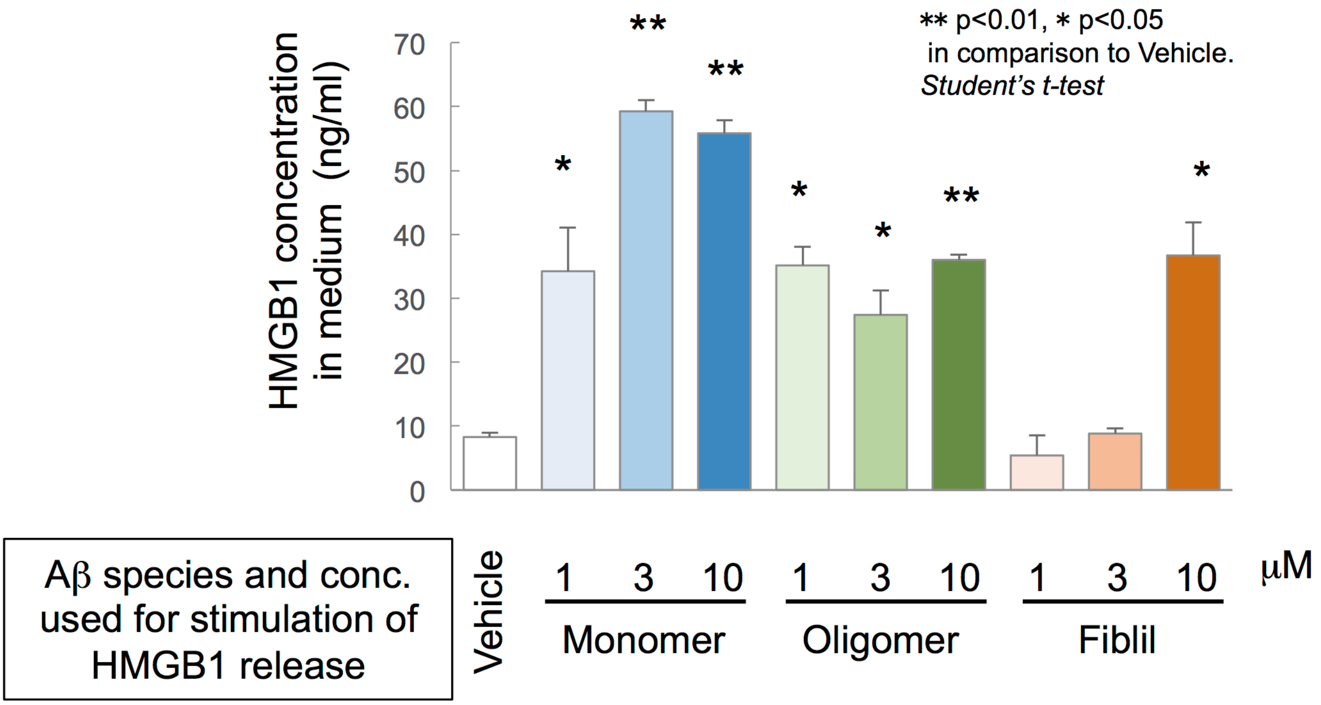

Supplementary Figure 19

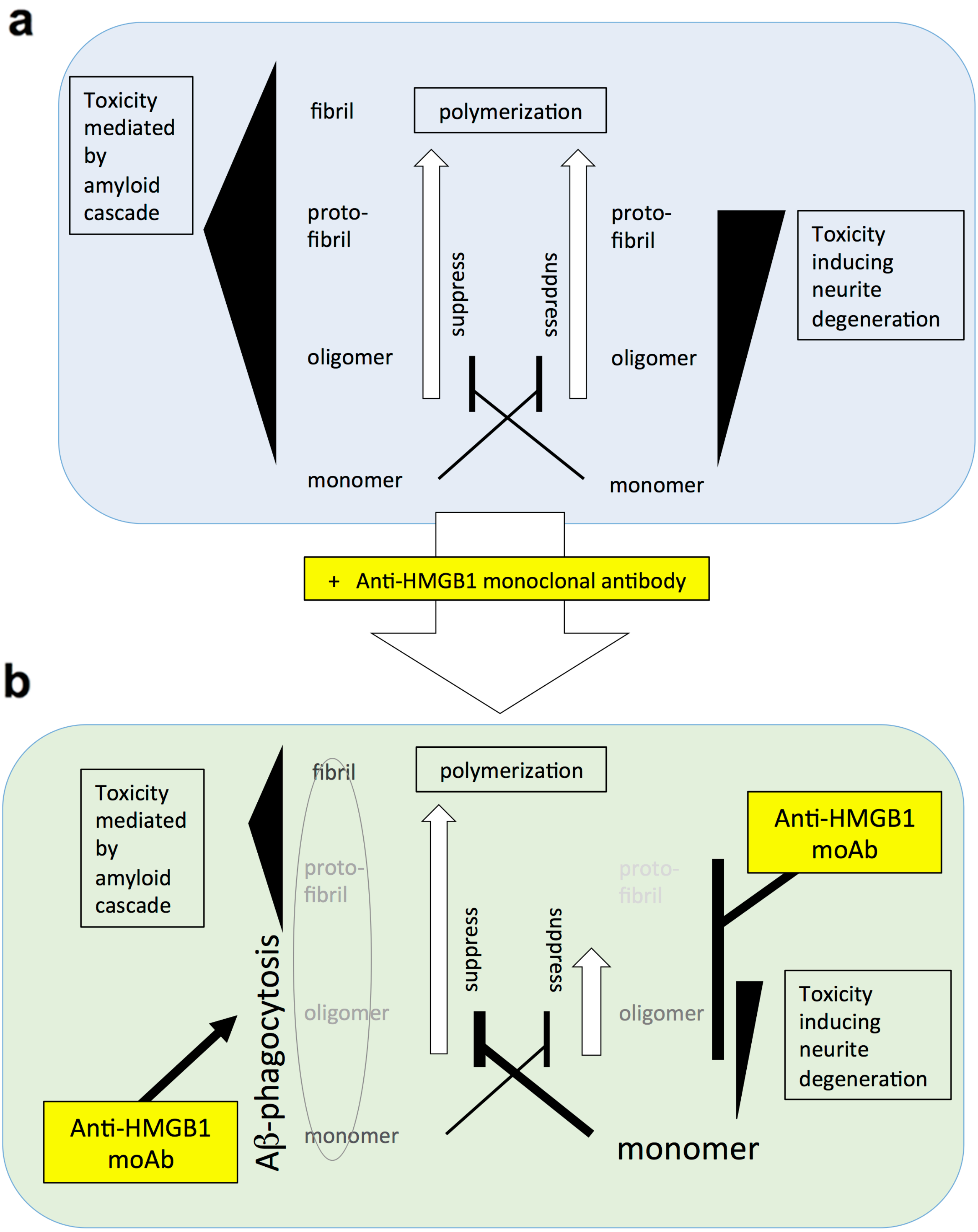

Supplement: Supplementary Information [file srep31895-s1.pdf]
